# Combinatorial Pooling Enables Selective Sequencing of the Barley Gene Space

**DOI:** 10.1371/journal.pcbi.1003010

**Published:** 2013-04-04

**Authors:** Stefano Lonardi, Denisa Duma, Matthew Alpert, Francesca Cordero, Marco Beccuti, Prasanna R. Bhat, Yonghui Wu, Gianfranco Ciardo, Burair Alsaihati, Yaqin Ma, Steve Wanamaker, Josh Resnik, Serdar Bozdag, Ming-Cheng Luo, Timothy J. Close

**Affiliations:** 1 Department of Computer Science and Engineering, University of California, Riverside, California, United States of America; 2 Department of Computer Science, Università di Torino, Torino, Italy; 3 Department of Botany and Plant Sciences, University of California, Riverside, California, United States of America; 4 Monsanto Research Centre, Bangalore, India; 5 Google, Inc., Mountain View, California, United States of America; 6 Joint Center for Genomic Research, King Abdulaziz City for Science and Technology, Riyadh, Saudi Arabia; 7 Department of Mathematics, Statistics and Computer Science, Marquette University, Milwaukee, Wisconsin, United States of America; 8 Department of Plant Sciences, University of California, Davis, California, United States of America; University of Tokyo, Japan

## Abstract

For the vast majority of species – including many economically or ecologically important organisms, progress in biological research is hampered due to the lack of a reference genome sequence. Despite recent advances in sequencing technologies, several factors still limit the availability of such a critical resource. At the same time, many research groups and international consortia have already produced BAC libraries and physical maps and now are in a position to proceed with the development of whole-genome sequences organized around a physical map anchored to a genetic map. We propose a BAC-by-BAC sequencing protocol that combines combinatorial pooling design and second-generation sequencing technology to efficiently approach *denovo* selective genome sequencing. We show that combinatorial pooling is a cost-effective and practical alternative to exhaustive DNA barcoding when preparing sequencing libraries for hundreds or thousands of DNA samples, such as in this case gene-bearing minimum-tiling-path BAC clones. The novelty of the protocol hinges on the computational ability to efficiently compare hundred millions of short reads and assign them to the correct BAC clones (*deconvolution*) so that the assembly can be carried out clone-by-clone. Experimental results on simulated data for the rice genome show that the deconvolution is very accurate, and the resulting BAC assemblies have high quality. Results on real data for a gene-rich subset of the barley genome confirm that the deconvolution is accurate and the BAC assemblies have good quality. While our method cannot provide the level of completeness that one would achieve with a comprehensive whole-genome sequencing project, we show that it is quite successful in reconstructing the gene sequences within BACs. In the case of plants such as barley, this level of sequence knowledge is sufficient to support critical end-point objectives such as map-based cloning and marker-assisted breeding.

## Author Summary

The problem of obtaining the full genomic sequence of an organism has been solved either via a global brute-force approach (called whole-genome shotgun) or by a divide-and-conquer strategy (called clone-by-clone). Both approaches have advantages and disadvantages in terms of cost, manual labor, and the ability to deal with sequencing errors and highly repetitive regions of the genome. With the advent of second-generation sequencing instruments, the whole-genome shotgun approach has been the preferred choice. The clone-by-clone strategy is, however, still very relevant for large complex genomes. In fact, several research groups and international consortia have produced clone libraries and physical maps for many economically or ecologically important organisms and now are in a position to proceed with sequencing. In this manuscript, we demonstrate the feasibility of this approach on the gene-space of a large, very repetitive plant genome. The novelty of our approach is that, in order to take advantage of the throughput of the current generation of sequencing instruments, we pool hundreds of clones using a special type of “smart” pooling design that allows one to establish with high accuracy the source clone from the sequenced reads in a pool. Extensive simulations and experimental results support our claims.

## Introduction

The second generation of DNA sequencing instruments is revolutionizing the way molecular biologists design and carry out investigations in genomics and genetics. These new sequencing technologies (e.g., Illumina, ABI SOLiD) can produce a significantly greater number of reads at a fraction of the cost of Sanger-based technologies, but with the exception of Roche/454 and Ion Torrent (ABI) read lengths are only 50–150 bases. While the number (and to a lesser extent the length) of reads keeps increasing at each update of these instruments, the number of samples that can be run has remained small (e.g., two sets of eight independent *lanes* on the Illumina HiSeq). Since the number of reads produced by the instrument is essentially fixed, when DNA samples to be sequenced are relatively “short” (e.g., BAC clones) and the correspondence between reads and their source has to be maintained, several samples must be “multiplexed” within a single lane to optimize the trade-off between cost and sequencing depth. Multiplexing is traditionally achieved by adding a DNA barcode to each sample in the form of an additional (oligo) adapter, but this does not scale readily to thousands of samples. Although it is possible to exhaustively barcode such a number of objects [1], the procedure of preparing (and balancing in multiplexes) thousands to ten of thousands of barcoded libraries for sequencing is very labor-intensive and can be quite expensive. Additionally, the resulting distribution of reads for each barcoded sample can be severely skewed (see, e.g., [2], [3]), necessitating rounds of selective follow-up.

Here, we demonstrate that multiplexing can be achieved without exhaustive barcoding by taking advantage of recent advances in combinatorial pooling design (also known as *group testing*). The essence of this work is to significantly reduce the burden of library production, without severely compromising on the sequencing coverage of each BAC. Combinatorial pooling has been used previously in the context of genome analysis, but here we attempt to use it for *de novo* genome sequencing. Earlier works use a simple grid design that can be very vulnerable to noise and behaves poorly when several objects are positive; a simple grid design is also far from optimal in terms of the number of pools it produces [4]–[7]. Recent works use more sophisticated pooling designs in combination with second-generation sequencing technology [8]–[12]. The application domain of “DNA Sudoku” is the detection of microRNA targets in *Arabidopsis* and human genes [9], whereas [8], [10] are focused on targeted resequencing (i.e., when a reference genome is available). Pooling designs have also been used to recover novel or known rare alleles in groups of individuals [11], [12].

In our approach to *de novo* sequencing, subsets of non-redundant but overlapping genome-tiling BACs are chosen to form intersecting pools. Each pool is then sequenced individually on a fraction of a flowcell via standard multiplexing. Due to the short length of a BAC (typically 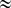
130 kb), cost-effective sequencing requires each sequenced sample to contain thousands of BACs. Assembling short reads originating from a mix of hundreds to thousands of BACs is likely to produce low-quality assemblies, as the assembler is unable to partition the reads according to individual BACs. Moreover, resulting contigs would not be assigned to a specific BAC. If instead reads could be assigned (or *deconvoluted*) to individual BACs, then the assembly could proceed clone-by-clone. We demonstrate that this objective can be achieved by choosing a pooling strategy in which each BAC is present in a carefully designed set of pools such that the identity of each BAC is encoded within the pooling pattern (rather than by its association with a particular barcode). We report experimental results on simulated data on the genome of *Oryza sativa* (rice) and real sequencing data on the genome of *Hordeum vulgare L*. (barley).

## Materials and Methods

**Figure 1:**
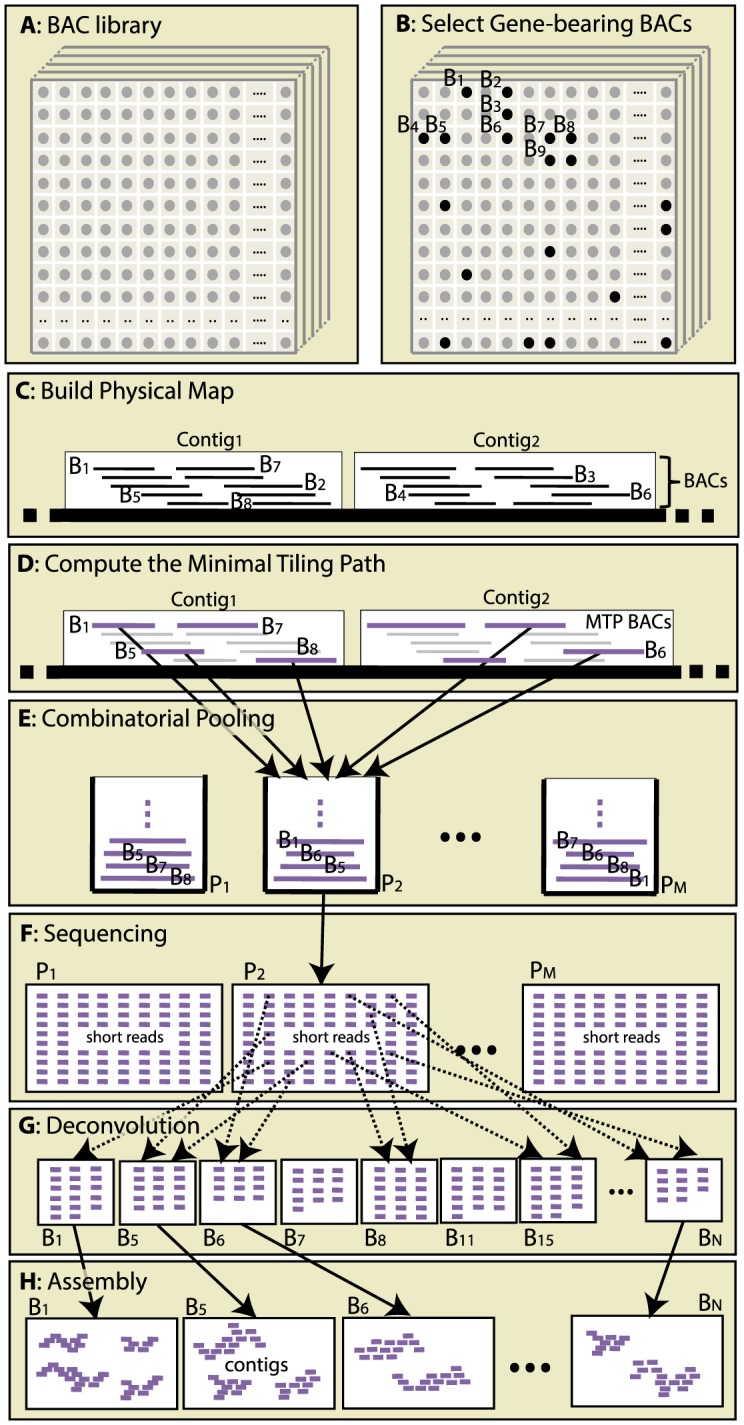
Proposed sequencing protocol. (A) obtain a BAC library for the target organism; (B) select gene-enriched BACs from the library (optional); (C) fingerprint BACs and build a physical map; (D) select a minimum tiling path (MTP) from the physical map; (E) pool the MTP BACs according to the shifted transversal design; (F) sequence the DNA in each pool, trim/clean sequenced reads; (G) assign reads to BACs (*deconvolution*); (H) assemble reads BAC-by-BAC using a short-read assembler.

The steps in our *combinatorial clone-by-clone sequencing* method are illustrated in Figure 1 and described next in detail.

**A.** Obtain a BAC library for the target organism

**B.** Select gene-enriched BACs from the library (optional)

**C.** Fingerprint BACs and build a physical map

**D.** Select a minimum tiling path (MTP) from the physical map [13], [14]

**E.** Pool the MTP BACs according to the shifted transversal design [15]

**F.** Sequence the DNA in each pool, trim/clean sequenced reads

**G.** Assign reads to BACs (*deconvolution*)

**H.** Assemble reads BAC-by-BAC using a short-read assembler

### Pooling minimum-tiling-path BACs (steps A–E)

While our method can in general be applied to any set of clones that cover a genome or a portion thereof, the protocol we describe here for selective genome sequencing uses a physical map of (gene-bearing) bacterial artificial chromosomes (BACs) to identify a set of minimally redundant clones. A *physical map* is a partial ordering of a set of genomic clones (usually BACs) encompassing one or more chromosomes. A physical map can be represented by a set of unordered contigs, where each contig is a set of overlapping clones. A physical map is usually obtained by digesting BAC clones via restriction enzymes into DNA fragments and then measuring the length of the resulting fragments (restriction fingerprints) on an agarose gel. The smallest set of clones that spans the region represented by the physical map is called *minimum tiling path* (MTP). The construction of a physical library and the selection of a MTP from a physical map are well-known procedures, and many organisms now have these resources available. More details can be found in, e.g. [14], [16]–[19], and references therein.

Once the set of clones to be sequenced has been identified, they must be pooled according to a scheme that allows the deconvolution of the sequenced reads back to their corresponding BACs. In Combinatorics, the design of a pooling method reduces to the problem of constructing a *disjunctive* matrix [20]. Each row of the disjunctive matrix corresponds to a BAC to be pooled and each column corresponds to a pool. Consider a subset 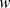 of the rows (BAC clones) in the disjunctive matrix, and let 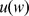 be the set of pools that contain at least one BAC in 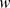. A design (or a matrix) is said to be 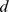-*decodable* if 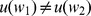 when 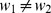, 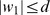, and 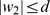. The construction of 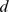-decodable pooling designs has been extensively studied [20]. The popular 2D grid design is simple to implement but cannot be used for the purposes of this work because it is only one-decodable.

Recently, a new family of “smart” pooling methods has generated considerable attention [8]–[10], [15], [21], [22]. Among these, we selected the *shifted transversal* design [15] due to its ability to handle multiple positives and its robustness to noise. The parameters of a shifted transversal design pooling are defined by three integers 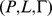, where 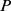 is a prime number, 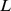 defines the number of layers, and 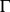 is a small integer. A *layer* is one of the classes in the partition of BACs and consists of exactly 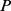 pools: the larger the number of layers, the higher is the decodability. By construction the total number of pools is 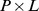. If we set 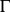 to be the smallest integer such that 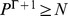 where 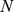 is the number of BACs that need to be pooled, then the decodability of the design is 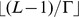. In [15], the shifted transversal design is defined by parameters 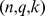 where 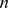 is the number of samples to be pooled, 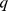 is the prime corresponding to the number of pools in each layer, and 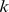 is the number of layers. In this paper, we use 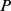 instead of 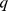, and 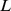 instead of 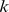.

An important property of this pooling design is that any two BACs share at most only 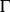 pools. By choosing a small value for 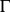 one can make pooling extremely robust to deconvolution errors. In our experiments, we use 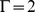, so that at least ten errors are needed to mistakenly assign a read to the wrong BAC. In contrast, two errors are sufficient to draw an erroneous conclusion with the 2D grid-design.

Barley BAC pools were obtained as follows. *Escherichia coli* strain DH10B BAC cultures were grown individually in 96-well plates covered by a porous membrane for 36 hr in 2YT medium with 0.05% glucose and 30 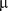
g/ml chloramphenicol at 37°C in a shaking incubator. Following combinatorial pooling of 50 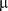
l aliquots from each of 2197 BAC cultures, each of 91 collected pools (169 BACs, 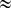
8.3 ml each) was centrifuged to create cell pellets. The pellets were frozen and then used for extraction of BAC DNA using Qiagen plasmid DNA isolation reagents. Each BAC pool DNA sample was then dissolved in 225 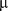
l of TE buffer at an estimated final concentration of 20 ng/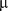
l. For gene-BAC assignment using the Golden Gate assays, a total of 10 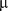
l (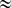
200 ng) of this DNA was then digested for 1 hour at 37°C by using 2 units of *Not*I enzyme with 100 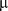
g/ml BSA in a volume of 100 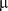
l. The *Not*I enzyme was then heat inactivated at 65°C for 20 min.

DNA for three sets of BACs (HV4,HV5, HV6) were prepared using a procedure that yields on average about 60% BAC DNA and 40% *E. coli* DNA. DNA for set HV3 was prepared using a procedure that was expected to yield about 90% BAC DNA and 10% *E. coli* DNA. Although these DNA samples performed well for SNP locus detection in the GoldenGate assay, we were unaware of the extent of *E. coli* in the samples until we began BAC pool sequencing, after all BAC pool DNAs had been prepared. The HV3 set reported here replaced an earlier set containing more *E. coli* DNA. Attempts were made to remove *E. coli* DNA from the BAC DNA samples through selective digestion by using exonucleases, and to reduce highly repetitive DNA using a denaturation/renaturation and double strand nuclease method. These procedures provided little or no reduction of the proportion of *E. coli* DNA in the samples. A cost-benefit analysis determined that the cost of replacing all of the BAC pools by applying an alternative BAC DNA purification procedure yielding an average of 90–92% BAC DNA and 8–10% *E. coli* DNA would be no more advantageous than simply repeating the sequencing of samples for which more DNA sequence information was needed to support the sequence-to-BAC deconvolution.

A video showing 76 seconds of the pooling process is available as Video S1.

### Sequencing and processing of paired-end reads (step F)

Sequencing of the barley BAC pools was carried out on an Illumina HiSeq 2000 at UC Riverside. Paired-end reads from each pool were quality-trimmed using a sliding window and a minimum Phred quality of 23. Next, Illumina PCR adapters were removed with Far (Flexible Adapter Remover, http://sourceforge.net), and the remaining sequence discarded either if shorter than 36 bases or if containing any ‘N’. Finally, reads were cleaned of *E. coli* (DH10B) and vector contamination (pBeloBAC11) using BWA [23] and custom scripts.

According to our simulations, the sequencing depth of each BAC after deconvolution is required to be at least 50x to obtain good BAC assemblies. The parameters of the pooling design should be chosen so that the coverage pre-deconvolution is at least 150x–200x to compensate for non-uniformity in the molar concentrations of individual BACs within each pool, and for losses due to sequencing errors.

### Deconvoluting reads to BACs (step G)

**Figure 2:**
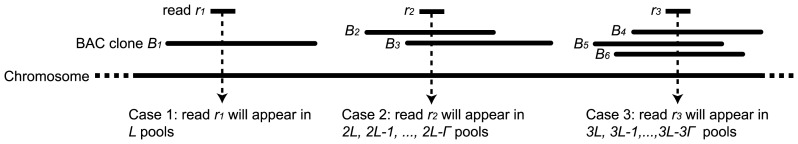
An illustration of the three cases we are dealing with during the deconvolution process (clones belong to a MTP).

To understand how deconvolution is achieved, let us make the simplifying assumption that clones in the MTP do not overlap. i.e., that the MTP BACs form a non-redundant tiling for the genome under study, or a fraction thereof. Let us pool the MTP BACs according to a shifted transversal design with 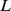 layers and obtain a set of reads from them. Now, consider a read 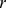 occurring only once in the portion of the genome covered by the BACs. If there are no sequencing errors and sequencing depth is sufficient, 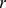 will appear in the sequenced output of exactly 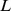 pools (see Figure 2, case 1). To determine the BAC to which read 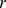 should be assigned, a search is made for the BAC signature that matches the list of positive pools for 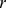.

For the most realistic scenario where at most 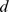 MTP clones overlap, the pooling must be at least 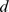-decodable for the deconvolution to work. We expect each non-repetitive read to belong to at most two BACs if the MTP has been computed perfectly, or rarely three BACs when considering imperfections, so we set 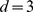. When a read belongs to the overlap between two clones (again assuming no sequencing errors), it will appear in the sequenced output for 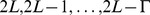 pools (see Figure 2, case 2). The case for three overlapping clones (see Figure 2, case 3) is analogous.

Recall that in step **E** each BAC is assigned to 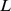 pools, thus the *signature* of a BAC is a set of 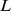 numbers in the range 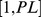, where the first number belongs to the range 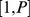, the second belongs to 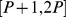,…, and the last one belongs to 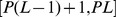. In our pooling design two BAC signatures cannot share more than 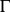 numbers (see Theorem I in [15]). One can think of BAC signatures as 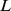-dimensional vectors which are rather “far” from each other.

Our deconvolution method proceeds as follows. First, recall the notion of *k-mer* of a string (read) 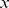 as a contiguous substring of 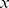 of length 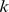. If we denote by 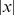 the length of string 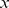, observe that 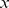 has 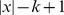 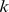-mers, not necessarily distinct. Let us call 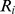 the set of reads obtained by sequencing pool 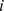, for all 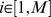, where 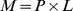 (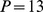 and 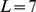 in our pooling design). For each set 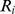, we first compute the number of occurrences 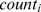 of each of its distinct 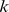-mers. Specifically, for each 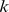-mer 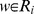 (i.e., for each 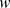 appearing in a read 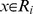), 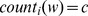 if 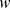 or its reverse complement occurs exactly 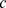 times in 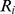. These counts are stored in a hash table such that, given a 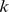-mer 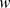, we can efficiently retrieve a count vector of 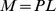 numbers, namely 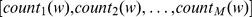. Once the table is built, we process each read as follows. Given a read 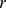 in pool 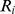, we fetch the count vectors for each of its 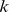-mers 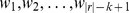. Given a 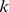-mer 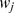, where 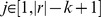, let 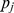 be the number of positive (non-zero) entries in its count vector 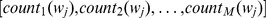, i.e., the number of pools where 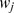 occurs at least once. Several scenarios are possible:

1. If 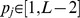, then 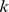-mer 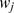 is discarded (it is likely to contain a sequencing error).
2. If 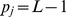 and if the set of 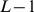 positive entries is a subset of one BAC signature, then 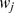 is assigned to the corresponding BAC.
3. If 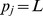 and if a perfect match between the set of 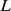 positive entries and a BAC signatures is found, then 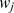 is assigned to the corresponding BAC.
4. If 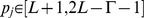, then the smallest 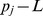 counts (other than the 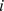-th one) are dropped from the count vector, and the new count vector with 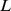 non-zero counts is handled by step 3.
5. If 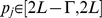 and if a perfect match between the set of positive entries and the union of two BAC signatures is found, then 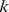-mer 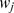 is assigned to the corresponding two BACs.
6. If 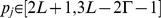, then the smallest 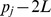 counts (other than the 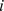-th one) are dropped from the count vector, and the new count vector with 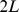 non-zero counts is handled by step 5.
7. If 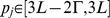, and if a perfect match between the set of positive entries and the union of three BAC signatures is found, then 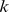-mer 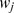 is assigned to the corresponding three BACs.
8. If 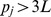, then 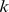-mer 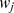 is discarded (it is highly repetitive).

At the end of this process, we consider the subset of 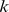-mers that have been assigned to one, two or three BACs and compute the union of their signatures, which becomes the *signature* of read 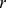. If a perfect match between the read signature and BAC signatures (either one, or the union of two or three) is found, then the read is assigned to the corresponding BAC(s). Reads for which no such match is found are declared *non-deconvolutable* and saved in a separate file.

This algorithm is implemented in the tool HashFilter, which has been extensively tested under Linux and MacOS. Source code and manual can be downloaded from http://www.cs.ucr.edu/stelo/hashfilter/, under the GNU General Public License.

### Clone-by-clone assembly (step H)

Once the reads have been assigned to individual BACs, sets of single and paired-end reads are assembled clone-by-clone using Velvet [24]. Velvet requires an expected coverage, which can be computed using the amount of sequenced bases assigned to each BAC and the estimated BAC size. For barley, BAC sizes were estimated from the number of bands in the restriction fingerprinting data. First, we computed the average number of bands in the 72,055 BACs fingerprinted using high-information-content fingerprinting [16], [17] (see also http://phymap.ucdavis.edu/barley/). Assuming that the average BAC length in this set was 106 kb, we computed the multiplier to apply to the number of bands to obtain the estimated BAC length, which turned out to be 1175 bases. We used that constant to obtain estimated sizes for all BACs in HV3, HV4, HV5 and HV6 (see Dataset S4). Note that the average size is over 125 kb, much larger than the library average size of 106 kb; this indicates that the MTP selection favors larger BACs.

We also tested SOAPdenovo [25] and Abyss [26] on simulated data, but there were no obvious performance benefits compared to Velvet in terms of assembly quality (data not shown). We evaluated the assembly for several choices of the 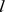-mer (hash) size, but have reported only the assembly that maximized the N50 (N50 indicates the minimum length of all contig/scaffolds that together account for at least 50% of the genome). We recorded the number of contigs, their N50/median/max/sum statistics, and the number of reads used in the assembly.

For rice assemblies, we Blast-ed the BAC contigs to the rice genome. We computed the fraction of the original (source) BAC covered by at least one contig, and the number of gaps and overlaps in the assembly. The parameters used for Blast are reported in the legend of Dataset S4.

For barley BAC assemblies, we carried out a validation based on the known BAC-unigene associations from the Illumina GoldenGate assay described in the next section. The validation involved Blast-ing EST-derived unigenes (Harvest:Barley assembly #35 unigenes, http://harvest.ucr.edu) against the BAC assemblies. To reduce spurious hits, we applied three filters. First, we masked highly repetitive regions by computing the frequency of all distinct 26-mers in the cleaned/trimmed HV5 data, then masking any 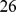-mers that occurred at least 11,000 times in the reads used for the assembly (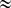
80 copies in the genome) from the assembled contigs, by replacing the occurrences of those repetitive 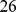-mers with Xs. Second, we ignored any BAC contig that covered a unigene for less than 50% of its length. Third, we excluded from the hit count any unigene that hit more than ten individual BACs overall. We recorded the number of unigenes hitting a BAC, and compared them with the expected unigenes according to the Illumina assay.

### Barley GoldenGate oligonucleotide pool assay

Samples for the GoldenGate assay were prepared by combining 5 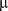
l of *Not*I-digested BAC pool DNA (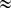
10 ng) with 4 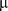
l of sonicated *E. coli* DNA pre-dialyzed into TE buffer at a concentration of 500 ng/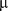
l (2000 ng) and 16 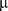
l of TE buffer. The final volume of each sample was thus 25 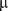
l, composed of 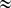
0.4 ng/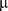
l of digested BAC pool DNA and 80 ng/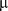
l of additional *E. coli* DNA. These DNA samples were provided to Joe DeYoung at the University of California, Los Angeles, California, or to Shiaoman Chao at the US Department of Agriculture genotyping facility in Fargo, North Dakota. The DNA concentrations were then readjusted to 50 ng/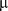
l and a total of 5 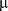
l of each DNA sample was used for each GoldenGate assay.

Each Illumina GoldenGate oligonucleotide pool assay (OPA) allows interrogation of a DNA sample for the presence of 1536 SNP loci. In [27], five OPAs were designed from approximately 22,000 SNPs from EST and PCR amplicon sequence alignments. Details of the development of three test phase (POPA1, POPA2, and POPA3) and two production scale (BOPA1 and BOPA2) can be found in [27]. We genotyped the barley BAC pools described in Section “The gene space of barley” on BOPA1 and BOPA2. The output from the Illumina GoldenGate assay was first converted to binary data by visual inspection of the theta/R space in BeadStudio. A positive reading meant that the SNP locus (and its corresponding unigene) is present in at least one BAC within the pool (refer to Figure S1 for an example).

Given the genotyping data for all unigene-pool pairs, we designed an algorithm that computes the optimal assignment of unigenes to BACs so that the number of errors is minimized. For a particular unigene 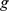 under consideration, let 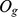 be the signature set of corresponding positive pools. Let 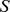 be an arbitrary set of BACs, where 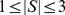, and 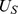 be the union of the pools that contain at least a BAC clone in 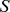. The number of errors 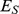 associated with this particular choice of 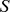 is defined to be the number of extra observations (equal to 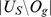) plus the number of missing observation (equal to 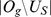). Among all possible choices of 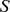, we chose 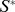 such that the value of 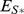 is minimized. When the number of errors associated with the final solution was too large (more than three), we declared that unigene to be *non-decodable*.

This procedure resulted in 1849 unigenes mapped to one, two, or three BACs. As a verification step, when a unigene was mapped to more than one BAC, we checked whether all those BACs belonged to the same contig in the barley physical map [18], [19]. Using the genetic map developed in [27], [28] we were also able to assign these unigene-anchored BACs to genetic map positions (Dataset S6) and to check whether a BAC was associated with more than one genetic map position. The unigene-BAC error rate from these cross-checking methods appeared to be about 5%.

### Barley whole genome shotgun sequencing

The whole genome shotgun sequencing of barley was carried out at several locations: Ambry Genetics (Aliso Viejo, California) sequenced five (2
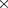
77 bases) paired-end lanes and four long-insert paired-end (LIPE) lanes (insert size of 2, 3 and 5 kb); University of Minnesota (courtesy of G. Muehlbauer) sequenced two (2
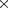
100 bases) paired-end lanes; UC Riverside sequenced seven (2
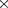
100 bases) paired-end lanes. The number of usable paired-end bases after quality-based trimming was 159.31 Gb and 4.92 Gb of LIPE, for an overall 31x sequencing depth of the 5.3 Gb barley genome. An 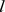-mer (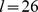) analysis showed that the effective depth of coverage of the data was about 24x [29].

## Results

### Simulation results on the rice genome

The physical map for *Oryza sativa* was assembled from 22,474 BACs fingerprinted at AGCoL, and contained 1,937 contigs and 1,290 singletons. From this map, we selected only BACs whose sequence could be uniquely mapped to the rice genome. We computed an MTP of this smaller map using our tool FMTP [14]. The resulting MTP contained 3,827 BACs with an average length of 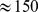 kb, and spanned 91% of the rice genome (which is 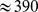 Mb). The overlap between rice BACs is significant: 1555 BACs overlap another BAC by at least 50 Kb, and 421 BACs overlap another BAC by at least 100 Kb (see Figure S2). In general, our method makes no assumption on the shared sequence content for pooled BACs.

We pooled *in silico* a subset of 2,197 BACs from the set above according to the shifted transversal design [15]. This pooling design is defined by three parameters 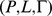 (see Materials and Methods for a detailed description of the properties of the pooling design). First observe that if the MTP was truly a set of minimally overlapping clones, a two-decodable pooling design would be sufficient. We decided that a three-decodable pooling scheme would give additional protection against errors and imperfections in the MTP. Taking into consideration the format of the standard 96-well plate and the need for a 3-decodable design, we chose parameters 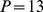, 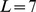 and 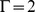, so that 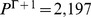 and 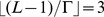. Each of the 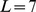 layers consisted of 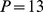 pools, for a total of 91 BAC pools, which left some space for a few control DNA samples on a 96-well plate. In this pooling design, each BAC is contained in 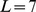 pools and each pool contains 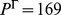 BACs. We call the set of 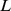 pools to which a BAC is assigned, the *BAC signature*. Any two BAC signatures can share at most 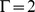 pools, and any triplet of BAC signatures can share at most 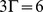 pools. Specifically, 57.9% of any BAC signature pairs have no pool in common, 30.6% share one pool, and 11.5% share two pools. For triplets of BAC signatures, 18.5% have no pool in common, 32% share one pool, 29.6% share two pools, 14.8% share three pools, 4.5% share four pools, 0.6% share five pools, and 0.01% share six.

The 91 resulting rice BAC pools were “sequenced” *in silico* by generating 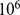 paired-end reads of 104 bases with an insert size of 327 bases, and 1% sequencing error distributed uniformly along the read. A total of 208 M usable bases gave an expected 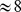
x sequencing depth for a BAC in a pool. As each BAC is present in seven pools, this is an expected 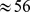
x combined coverage.

The 91 read pools were processed for deconvolution using the 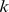-mer based algorithm presented in the Materials and Methods section. We set 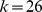 because we wanted to detect an overlap between two reads of 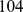 bases with a length of at least 75% (78 bases) and at most two mismatches (observe that if the two errors are equally spaced along the 78 overlapping bases, a perfect match of length 26 must occur). The computation was relatively quick, but required a significant amount of memory. The construction of the hash table required about 120 GB of RAM and 164 minutes running on one core of a Dell PowerEdge T710 server (dual Intel Xeon X5660 2.8 Ghz, 12 cores, 169 GB RAM). The deconvolution phase took 33 minutes on 10 cores; sorting the reads into 2,197 files took 22 minutes on one core.

**Figure 3:**
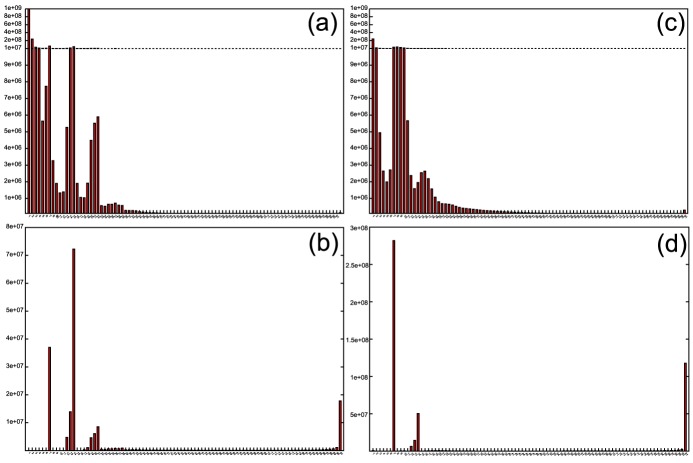
Count distribution for the signatures of all distinct 26-mers [(a) rice synthetic data, (c) barley HV5] and all the reads [(b) rice synthetic data, (d) barley HV5] in the 91 pools of sequencing data. The x-axis represents the size of the signature and the y-axis is the absolute count.

Figure 3-(a) illustrates the distribution of signature sizes for all the distinct 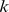-mers in the rice dataset. Observe that the distribution has clear peaks around 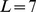, around the interval 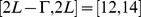 and the interval 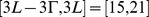. These peaks correspond to 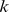-mers originating from one, two, and three overlapping BACs, respectively (see Figure 2). We also have a rather large number of 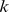-mers appearing in 1–6 pools. Observe that if the sequencing depth was sufficient, and in the absence of technical errors with BACs for a long 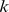-mer to have fewer than 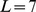 occurrences, sequencing errors must have occurred. Figure 3-(b) shows the distribution of signature sizes for all the reads in the rice dataset at the outset of our deconvolution algorithm (presented in the Materials and Methods section). Observe that the vast majority of reads now have a signature size in the expected ranges, with the exception of reads that appear in more than 80 pools. This latter set of reads cannot be deconvoluted and is discarded.

The set of reads with a signature of size 7, 12–14 or 15–21 that could be deconvoluted was 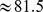
% of the total (see Table S2 and Dataset S1). Since we knew the BAC from which each read was generated, we determined that 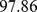
% of the deconvoluted reads were assigned to either the correct BAC or to a BAC overlapping the correct BAC (see Table S2 and Dataset S4). After deconvolution, the average sequencing depth for each BAC was 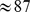
x, about 50% higher than the expected 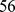
x. Even if we are losing about 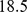
% of the reads due to invalid signatures, deconvoluted reads are frequently assigned to multiple BACs, thereby amplifying the sequencing depth. Part of this inflation can be attributed to the overlap between BACs in the rice MTP (see Figure S2).

In the final step of the protocol, we independently assembled the set of reads assigned to each BAC. We carried out this step with Velvet [24] for each of the 2,197 BACs, for a variety of choices of 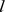-mer size (hash length) and reported only the assembly that maximized the N50. This is an arbitrary choice that does not guarantee the “best” overall assembly. Sheet 1 in Dataset S4 summarizes the results. If we average assembly statistics over all the 2,197 BACs, the percentage of reads used in the assembly was 82.3%, the average number of contigs was 41, the average N50 was 47,551 bp (31.4% of the average BAC length), the average largest contig was 57,258 bp (37.8% of the average BAC length), the average sum of all contig sizes was 137,050 bp (90.7% of the average BAC length). The N50 is quite high, and so is the percentage of reads used by the assembler. While these numbers already indicate high quality assemblies, we determined whether BACs were correctly assembled by Blast-ing BAC contigs against the rice genome. Sheet 1 in Dataset S4 reports the results of this analysis. Considering these statistics over all the 2,197 BACs, the average BAC coverage was 76.8%, the average gap size was 263 bp, the average number of gaps was 138, the average overlap size was 107 bp, and the average number of overlaps was 75.

To establish a comparison “baseline” for these assembly statistics, we considered the most optimistic scenario of a “perfect deconvolution”, which entails using the provenance annotation of each read to assign it back to the correct BAC with 100% accuracy. Sheet 2 in Dataset S4 reports the same statistics for all 2,197 BAC assemblies in this best-case scenario. If we compute the average over all the 2,197 BACs, the average fraction of the reads used by Velvet was 82.7% and the average N50 was 132,865 bp (88% of the average BAC length). The Blast statistics showed an average BAC coverage of 96.3%, an average gap size of 52 bp, an average number of gaps of 97, an average overlap size of 29 bp, and an average number of overlaps of 54. While this latter BAC coverage is about 20% higher, the results following deconvolution compare quite favorably with what would be possible sequencing each BAC separately.

### The gene-space of barley

Barley's diploid genome size is estimated at 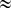
5,300 Mb and is composed of at least 80% highly repetitive DNA, predominantly LTR retrotransposons [30]. We started with a 6.3x genome equivalent barley BAC library which contains 313,344 BACs with an average insert size of 106 kb [31]. Nearly 84,000 gene-enriched BACs were identified, mainly by the overgo probing method [32]. Gene-enriched BACs were fingerprinted using high-information-content fingerprinting [16], [17]. From the fingerprinting data a physical map was produced [18], [19] and a MTP of about 15,000 clones was derived [14]. Seven sets of 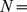
2,197 clones were chosen to be pooled according to the shifted transversal design [15], which we internally call HV3, HV4,…, HV9 (HV1 and HV2 were pilot experiments). We used the same pooling parameters discussed in the previous section (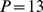, 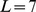 and 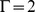).

Here we are reporting on four out of seven sets, namely HV3, HV4, HV5, and HV6. Each is comprised of 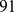 pools with a total of 2,197 MTP gene-rich barley BAC clones. Given the estimated 129.5 kb size of a BAC in the barley MTP (see Materials and Methods), the total complexity of each pool of 169 BACs is 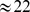 Mb and of the 2,197 BACs is 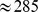 Mb. To take advantage of the high density of sequencing of the Illumina HiSeq2000, 13–16 pools were multiplexed on each lane, using custom multiplexing adapters.

After each sample was sequenced and the reads demultiplexed, we obtained an average of 22.9 M, 11.3 M, 11.5 M, and 10.1 M reads per pool in HV3, HV4, HV5, and HV6, respectively, with a read length of 92 bases. Reads were quality-trimmed and cleaned of spurious sequencing adaptors, and then reads derived from *E. coli* contamination or the BAC vector were discarded (see Dataset S2). The percentage of *E. coli* contamination was rather high, averaging around 41%, 40%, 51%, 65% in HV3, HV4, HV5, and HV6, respectively. An alternative DNA purification method we used for HV3 showed the potential to lower amount to 8–15% if properly executed (see some of HV3 pools in Dataset S2, column I). The average number of usable reads after trimming and cleaning was about 13.5 M, 6.8 M, 5.5 M, and 3.6 M per pool in HV3, HV4, HV5, and HV6, respectively, with an average high quality read length of 87–89 bases. The number of reads in the set of 91 pools ranged between about 4.2 M–27.7 M in HV3, 2.9 M–17 M in HV4, 2.5 M–11.3 M in HV5, and 1.6 M–15 M in HV6. The total number of reads was about 1229 M, 620 M, 503 M, and 327 M in HV3, HV4, HV5, and HV6, respectively, for a total of about 109.1T, 54.9T, 44.8T, and 28.5T usable bases, respectively.

The 91 read pools in the barley datasets were processed using the 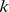-mer based algorithm (HashFilter with 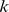 = 26) described in the Materials and Methods section. The computation took slightly longer than on the analogous rice dataset (i.e., about 363 minutes on one core of a Dell PowerEdge T710 server to build the hash table for HV5), but used less memory (i.e., about 43 Gb of RAM for HV5). For HV5, the deconvolution phase took 169 minutes on 10 cores, and the sorting of reads into 2,197 BAC files took 37 minutes on one core. Due to the higher repeat content of the barley genome compared to rice, we were able to deconvolute a smaller fraction of the barley reads, about 68.14% for HV3, 59.9% for HV4, 71.3% for HV5, and 58% for HV6 (see Tables S3, S4, S5, S6 and Dataset S3). Figure 3-(c) and (d) illustrate the distribution of signature sizes for all the distinct 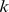-mers and for all reads in HV5. As expected, the number of reads occurring in over 80 pools is much higher for barley than for rice. After deconvolution, the total number of usable bases was about 97.9T for HV3 (about 90% of the bases before deconvolution), 34.6T for HV4 (about 63%), 38.9T for HV5 (about 87%), and 19.3T for HV6 (about 68%), which translated to an average sequencing depth of coverage for each BAC of about 431x in HV3, 134x in HV4, 137x in HV5, and 72x in HV6 (see Dataset S4).

On the set HV5, we tested HashFilter for different choices of the 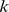-mer. For 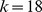, 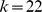, and 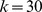, the memory used by HashFilter was about 35 GB, 40 GB, and 48 GB, respectively (compared to 43 Gb of RAM for 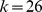). On one CPU core, the time to build the hash table was about 323 minutes for 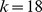, 338 minutes for 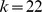, and 915 minutes for 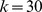, compared to 363 minutes for 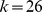. On ten CPU cores, the overlap phase took 108 minutes for 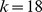, 165 minutes for 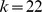, and 244 minutes for 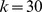, compared to 169 minutes for 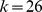. In terms of the number of reads deconvoluted, for 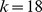, 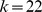, and 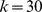, we deconvoluted 62.2%, 68.2%, and 73.6% of the reads in HV5, respectively (compared to 71.3% when 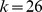 – see Dataset S3 for more details).

We have collected strong evidence that the deconvolution accuracy was quite high in barley. For instance, six BACs that were assigned less than 20 reads in HV5 were noted as not growing during the pooling (for a video of the pooling see Video S1). For two HV sets, we realized that we erroneously swapped two adjacent pools after noticing that the percentage of deconvoluted reads for those pools was significant lower than the average. Later we confirmed the swap by mapping the reads in those pools to the unigenes that were known to be present in the pooled BACs. During the same investigation, we also assessed how the overall percentage of deconvolution would be affected if one pool was missing. We removed all the reads in a pool of HV3, and re-executed the deconvolution algorithm: the number of deconvoluted reads decreased by less than 0.5%, a very small loss considering that an entire pool was removed. We also carried out an analysis of deconvoluted paired-end reads for HV5 to determine to what extent the left and the right mate agreed on their BAC(s) assignment. The deconvolution treated paired-end reads as two separate single-end reads, which were processed independently. For each paired-end read 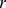, we collected in 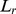 the set of BACs assigned to the left mate, and in 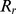 the set of BACs assigned to the right mate. Unless 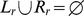, we declared the paired-end read 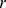 to be *concordant* when 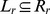 or 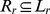. For barley HV5, 68.7% of the deconvoluted paired-end reads were concordant, which indicates that the deconvolution was quite accurate (see the second sheet in Dataset S3).

We assembled each set of reads assigned to a BAC using Velvet [24] for a variety of choices of 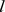-mer size. From the assemblies obtained for different choices of 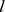, we decided to report in Dataset S4 the assembly that maximized the N50. If we average the assembly statistics over the 2,197 BACs, the number of deconvoluted reads used in the assemblies was 84% in HV3, 86% in HV4, 87.6% in HV5, and 83.2% in HV6 indicating that Velvet took advantage of most of the data; the average N50 was 8,190 bp in HV3 (7.0% of the average BAC length in that set), 5,883 bp in HV4 (4.65% of the average BAC length), 7,210 bp in HV5 (5.6% of the average BAC length), and 6,032 in HV6 (4.67% of the average BAC length); the average longest contig was 18,958 bp in HV3, 15,674 bp in HV4, 19,222 bp in HV5, and 16,018 in HV6; the average sum of all the contigs in each assembly was 104,578 bp in HV3 (89.8% of the average BAC length in that set), 102,502 bp in HV4 (81.5% of the average BAC length), 113,678 bp in HV5 (87.8% of the average BAC length), and 98,087 bp in HV6 (75.9% of the average BAC length).

Barley BAC assemblies were compared against BAC-unigene lists obtained using the Illumina GoldenGate oligonucleotide pool assay (OPA) [33] developed for barley [27]. We used the Illumina OPAs on the same four, and three additional, sets of barley pools described above (637 pools in total) and determined which BAC clones were positive for two sets of 1,536 marker loci/unigenes (see Materials and Methods for details). The Illumina OPAs allowed us to map a total of 1,849 unique unigenes to BACs (estimated error rate of 5%, see Materials and Methods for details). Table S1 summarizes the results of unigene-BAC and BAC-unigene assignment broken down by chromosome and chromosome arms, whereas Dataset S6 contains all the solved BAC-unigene relationships along with their chromosomal location.

Analysis of the assembly of the 2,197 barley BACs in each of the HV3–HV6 sets was carried out by assuming the results of the OPA as the “ground truth”, although the Illumina OPA assay has an estimated error rate of 5%. For instance in HV5 we extracted a total of 221 marker loci/unigenes that were mapped to a total of 202 distinct BACs. We obtained the sequence of these 221 unigenes from Harvest (http://harvest.ucr.edu) and Blast-ed them against the HV5 BAC contigs. Out of 202 BACs that were expected to contain those genes, only 20 BAC assemblies (10%) missed the expected marker loci/unigenes (see Dataset S4). For the other 90% of the assemblies which contained the expected unigenes, the average coverage of those unigenes was about 90% of their length. Similar results were obtained from the HV3, HV4, and HV6 sets (see Dataset S4). This analysis suggests that these BAC assemblies contain the majority of the barley genes, which is the main objective of this work.

## Discussion

The challenges of *de novo* sequence assembly originate from a variety of issues, but two are the most prominent. First, sequencing instruments are not 100% accurate, and sequencing errors in the form of substitutions, insertion, or deletions complicate the detection of overlaps between reads. Sequencing errors can be detected and potentially corrected when the sequencing depth is sufficient (see, e.g., [34]). Second, a very large fraction of eukaryotic genomes is composed of repetitive elements. During the assembly process, reads that belong to those repetitive regions get *over-compressed* which in turns lead to mis-assemblies [35]. To cope with the problems caused by repeats, two strategies have been proposed: *paired-end* and *clone-by-clone* sequencing. In paired-end sequencing, pairs of reads are obtained from both ends of inserts of various sizes [36], [37]. Paired-end reads resolve repeats during assembly simply by jumping across them (abandoning the effort to fully resolve them) and disambiguating the ordering of flanking unique regions. In clone-by-clone sequencing, chunks of the genome (100–150 kb) are cloned, typically in BACs, and then reads are obtained independently from each clone [38]. Sequences that are repetitive in the context of the whole genome are more likely to have only a single copy in a given BAC, which increases the fraction of the BAC that can be assembled. Sequencing technologies based on flow cells have significantly reduced the cost of of generating raw sequence data, but, with the exception of Roche/454 and Ion Torrent (each of which suffers from a homopolymer issue), the reads are much shorter than Sanger reads. Shorter read length makes the problem of *de novo* genome assembly significantly harder: the shorter a read is, the more likely it is repetitive in its entirety [35].

The major technical hurdle for a clone-by-clone approach is the limitation of these instruments in handling hundreds or thousand of BACs in a way that would allow reads to be assigned back to their source. DNA barcoding can be used (see e.g., [1]), but the process of preparing thousands of barcoded libraries for sequencing is very labor-intensive and expensive. Here, we have demonstrated an efficient alternative to barcoding: by encoding the “signature” of a BAC in the unique set of pools to which it is assigned, reads originating to that BAC will also share the same signature; this enables their deconvolution to the original BAC. We note that our deconvolution method does not require paired-end data, although such type of reads is desirable for the assembly.

Experimental results on simulated data for rice and actual sequencing data for barley show that our pooling-based clone-by-clone approach can be carried out effectively on short-read sequencing instruments. Our method deconvolutes reads to single BACs with very high accuracy (97.86% on rice), and as a consequence the assemblies of the resulting BAC clones are of high quality. For the synthetic data (containing 1% sequencing errors) on the rice genome, we were able to reconstruct on average 77% of the BAC content. On the barley data, we successfully assembled 90% of the expected unigenes, with an average coverage of the unigenes of nearly 90%. This amount of sequence is sufficient for a wide range of practical purposes such as map-based cloning and nearby marker development for marker-assisted breeding.

We are aware of the limitations of our approach, namely (1) the need for a physical map, (2) the significance of *E. coli* contamination, (3) the inability of the deconvolution algorithm to deal with repetitive reads (i.e., reads that appear in more than three BACs or twenty-one pools in our pooling design), and (4) the deleterious effect of sequencing errors on the deconvolution. Issue (1) can be quite limiting, although for many economically or ecologically important organisms several research groups and international consortia have produced clone libraries and physical maps. One can address (2) by alternative, more expensive BAC DNA purification procedures that are expected to reduce *E. coli* contamination to less than 10%, which we tested on the HV3 set with promising results. One should keep in mind, however, that this approach might not be more cost-effective than simply generating more sequencing data. Somewhat related to this problem is the variability in sequencing quality among pools: one of the anonymous reviewers suggested that one could use *E. coli* reads to establish a quality metric for the sequencing step of each individual pool of BACs. Regarding (3), we speculate that the removal of “ubiquitous” reads from the BAC assembly might not significantly affect the quality of the assembly, because even if these highly-repetitive reads were assigned to the correct BAC the assembler would not be able to assemble them. We do not have, however, data to support this claim. In the future we will consider assembling all reads in a pool that do not deconvolute, then adding the resulting contigs to the BAC contigs during the merging step. Finally, to address (4), observe that the presence of one sequencing error in a read affects at most 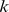 consecutive 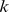-mers overlapping the error. These erroneous 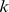-mers are likely to occur in a small number of pools (fewer than 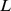): observe for instance in Figure 3-(a) and Figure 3-(c) a rather large number of 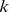-mers appearing in 1–6 pools. One could then design an error-correcting method that attempts to correct sets of consecutive 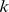-mers that appear in fewer than seven pools. Error-correction and deconvolution are mutually dependent: correcting the reads will help the deconvolution, which in turns will lead to further error-correction. Due to this mutual dependency, the accuracy of the deconvolution algorithm could be also improved by a multi-stage approach where one initially assign high-quality reads to BACs, then use these assignments to conservatively correct a portion of the reads, which in turns will allow to deconvolute more reads, and so forth, until a fixed point is reached. Additionally, the process of correcting the reads will simplify the assembly process, which is expected to produce more accurate assemblies.

To summarize the trade-offs between the target size (e.g., BACs, set of BACs, whole genome), sequencing depth, and the number/size of assembled contigs, we collected a set of critical assembly statistics in Table 1. The first two rows contain average BAC assemblies statistics for rice data, assuming perfect deconvolution or deconvolution via our tool HashFilter, then assembled with Velvet. Average assembly statistics for a single BAC of barley HV3–HV6 are reported on rows 5–8. The third and ninth row represent the average statistics obtained by assembling all the reads in each pool of 169 BACs of rice and barley HV5, respectively, using Velvet with the 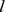-mer size that maximized the N50 (see Dataset S5). We wanted to use Velvet for all the assemblies because it can track reads and gives accurate statistics about the number of reads used in the assembly, but it cannot efficiently handle very large datasets of reads. For the other rows in the table we had to use SOAPdenovo [25], however Velvet and SOAPdenovo can be considered comparable in terms of assembly performance [39], [40]. The fourth and tenth row report the assembly of all the reads in the 91 pools for rice and barley HV5, respectively, using SOAPdenovo with 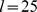. Finally, the last row reports the statistics of a 31x whole-shotgun assembly of the barley genome using SOAPdenovo with 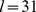 (see Materials and Methods for details about the data).

**Table 1:** Summary of the statistics of the various assemblies obtained using Velvet (rows 1–3, 5–9) and SOAPdenovo (rows 4, 10, 11).

*Target*	*Size* (Mb)	*Seq. depth*	*% reads used* c	*N50* (bp)	*% Sum*
Rice – 1 BAC (perfect deconvolution)^a^	0.151	56x	82.7%	132,865	98.7%
Rice – 1 BAC (HashFilter deconvolution)^a^	0.151	87x	82.3%	47,551	90.7%
Rice – 169 BACs (no deconvolution)^b^	26	56x	83.2%	4,236	73.1%
Rice – 2,197 BACs ( 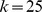 , no deconvolution)	332	56x	5.9%	1,148	30.6%
Barley HV3 – 1 BAC (HashFilter deconvolution)^a^	0.116	431x	83.6%	8,190	89.8%
Barley HV4 – 1 BAC (HashFilter deconvolution)^a^	0.125	134x	86.0%	5,883	81.5%
Barley HV5 – 1 BAC (HashFilter deconvolution)^a^	0.129	137x	87.6%	7,210	87.8%
Barley HV6 – 1 BAC (HashFilter deconvolution)^a^	0.129	72x	83.2%	6,032	75.9%
Barley HV5 — 169 BACs (no deconvolution)^b^	22	26x	67.1%	4,270	69.5%
Barley HV5 – 2,197 BACs ( 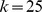 , no deconvolution)	286	180x	25.3%	3,845	56.6%
Barley – whole genome ( 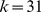 )	5,300	31x	13.3%	2,857	30.5%

“% Sum” is the the sum of all contig sizes as percentage of the target size;

a average over 2,197 assemblies;

b average over 91 assemblies;

c Velvet reports the number of reads used in the assembly but SOAPdenovo does not: for these assemblies we used Bowtie (allowing one mismatch) to align reads to the contigs.

An analysis of the statistics reported in Table 1 clearly show that as the complexity of the target sequence increases from one BAC to the whole genome, both the N50 and the percentage of reads used by the assembler decrease, as does the sum of all contig sizes as a fraction of the target size. This indicates that the effectiveness of the assembler decreases as the complexity of the assembly problem increases. Similar conclusions were reported in [41], where the assembly of pools of BACs were of significantly better quality than shotgun assemblies of *Arabidopsis*. While we are not advocating to abandon the whole genome shotgun approach in favor of clone-based sequencing, there is clearly an opportunity to combine the advantages of BAC-by-BAC and whole genome shotgun assemblies. This synergistic step could represent a significant advance in solving the problem of obtaining a high quality assemblies for large, highly repetitive genomes.

### Description of additional data files and software

Barley raw sequencing data for the barley BAC set can be obtained from NCBI Sequence Read Archive accession numbers SRA051771 and SRA051780 (HV3), SRA051535 (HV4), SRA047913 (HV5) and SRA050074 (HV6). When all the BAC assemblies will be complete, we will make them available in Harvest:Barley (http://harvest.ucr.edu) and GenBank (http://www.ncbi.nlm.nih.gov/genbank/). The current set of HV3, HV4, HV5, and HV6 BAC assembly as well as the 31x shotgun genome assembly of barley can be accessed via our Blast server hosted at the address http://www.harvest-blast.org/, by selecting “Morex Barley BACs” or “Barley Genome” from the database menu. These assemblies can also be downloaded from http://www.harvest-web.org/utilmenu.wc. The source code of HashFilter is available from http://www.cs.ucr.edu/stelo/hashfilter/under the GNU General Public License. HashFilter runs under Linux or MacOS.

Additional data are available with the online version of this paper.

## Supporting Information

Dataset S1Excel file Dataset_S1 contains the pool-by-pool statistics of the number of rice reads deconvoluted to one BAC (column C), two BACs (column D), and three BACs (column E), as well as the percentage of reads deconvoluted as a fraction of the total number of reads in each pool (column F). Columns G-K report the paired-end read analysis: for each paired-end read (total is column G), we looked at the set of BACs assigned to the left read (set 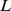) and the right read (set 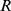) via deconvolution. The number of paired-end reads for which at least one of 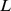 and 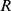 were non-empty is reported in column I. Of these, we compared 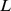 and 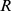: if the number of shared BACs in 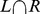 was equal to the 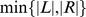 we declared that paired-end read *concordant*. Column J reports the number of concordant deconvoluted paired-end reads.(XLSX)

Dataset S2 Excel file Dataset_S2 contains the statistics of number of reads and bases at each step of the cleaning process for sets HV3, HV4, HV5 and HV6.
(XLS)

Dataset S3Excel file Dataset_S3 contains the pool-by-pool statistics of the number of barley HV3, HV4, HV5 and HV6 reads deconvoluted to one BAC (column C), two BACs (column D), and three BACs (column E), as well as the percentage of reads deconvoluted as a fraction of the total number of reads in each pool (column F). For the HV5 tab, columns G-L reports on the paired-end read analysis: for each paired-end read (total is column H – obtained by subtracting the single reads from each pool), we looked at the set of BACs assigned to the left read (set 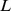) and the right read (set 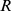) via deconvolution. The number of paired-end reads for which at least one of 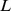 and 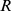 were non-empty is reported in column J. Of these, we compared 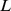 and 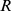: if the number of shared BACs in 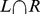 was equal to the 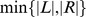 we declared that paired-end read *concordant*. Column K reports the number of concordant deconvoluted paired-end reads.(XLSX)

Dataset S4Excel file Dataset_S4 contains eight tables/sheets (two for rice, two tabs for barley HV3, two tabs for barley HV4, two for barley HV5, and two for barley HV6). The first sheet contains rice BAC data before and after deconvolution (columns A–H), assembly statistics (columns I–S), and Blast results (columns T–Y). Columns A–B are self-explanatory. Column C shows the list of pools where that BAC was assigned (called *BAC signature*). Column D shows the number of reads generated *in silico* for each rice BAC, column E is the actual size of the BAC, column F reports the number of reads deconvoluted to that BAC, column G the number of reads correctly deconvoluted, and column H the percentage of the reads correctly deconvoluted. A read is “correctly deconvoluted” if the list of BACs to which it has been assigned contains the BAC from where it was generated. Columns I–S report the results of running Velvet on the deconvoluted reads, BAC-by-BAC. For each BAC, ten choices of 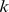-mer were tested, in the range [25,79] with a step of 6. The spreadsheet reports only the assembly that maximized the N50. This is an arbitrary choice that does not guarantee the “best” overall assembly. Column titled “K” shows the value of the 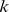-mer in the range which maximized the N50. Column “Cnt” shows the number of contigs produced by Velvet. Column “Used” reports the fraction of the reads used by Velvet. “Med”, “Mean”, “n50”, “max” show the median, mean, n50 and max contig size, respectively. Column “Sum” is the sum of the size of all contigs/scaffolds. “n50/Sz” and “Sum/Sz” report the size of the n50 and the sum over the expected BAC size. “Ns” report the total size of gaps in the scaffolds. Finally, columns T–Y report the results of Blast-ing the assembly (contigs) to the actual BAC sequences. Column “HSPs” is the number of high scoring segment pairs, column “Covg” shows the percentage of BAC covered by the contigs of the assembly, column “Gaps” reports the number of gaps (i.e., regions of the BAC not covered by any contig), column “AvgGap” is the average length of gaps, column “Overlaps” shows the number of regions where several HSPs overlap (i.e., portions of the BAC covered more than once), and column “AvgOvr” is the average length of the overlaps. The parameters used for Blast are: Expect threshold = 0.001, Align limit = 10, Best hit overhang = 0.1, and Best hit score edge = 0.1; for parsing the Blast results we used Align limit = 1, HSP limit = 10, and Min length = 100. The second sheet contains the assembly statistics for rice BAC data, assuming that reads are mapped to their original BAC with 100% yield and accuracy. Please refer to the previous description for an explanation of the column contents. The next six tables/sheets are two pairs each for HV3, HV4, HV5, and HV6, respectively. The sheet called “Assembly Stats” shows BAC and assembly statistics for each of the 2,197 BACS in the HV3–6 sets. The first set of columns (A–G) refer to the barley BAC stats. Columns A-C are self-explanatory (column B shows the list of pools where that BAC was assigned, called *BAC signature*). Column “Size Estim” is the expected BAC size, estimated from the number of bands in the fingerprinting data. “Reads” contain the number of reads mapped to that BAC, “Bases” is the total number of bases, and “Covg” is the expected coverage. The next set of columns (H–M) refer to Velvet's assembly statistics. For each BAC, ten choices of 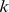-mer were tested in the range [25,79] with a step of 6. The spreadsheet reports only the assembly that maximized the N50. Column titled “K” shows the value of the 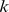-mer in the range which maximized the N50. Column “Cnt” shows the number of contigs produced by Velvet. Column “Used” reports the fraction of the reads used by Velvet. “Med”, “Mean”, “n50”, “max” show the median, mean, n50 and max contig size, respectively. Column “Sum” is the sum of the size of all contigs/scaffolds. “n50/Sz” and “Sum/Sz” report the size of the n50 and the sum over the expected BAC size. “Ns” report the total size of gaps in the scaffolds. The next set of columns (S–V) report the results of Blast-ing U35 unigenes (Harvest:Barley assembly #35 unigenes, http://harvest.ucr.edu) against the BAC assemblies. We used BlastN with e-value threshold 1e-20. To reduce spurious hits, we applied three filters (see Materials and Methods for details). Column “Hits” shows the number of unigenes hitting that BAC – note that some BACs can be hit by a set of unigenes from a multi-gene family so the count can be high. For a small set of BACs, we knew in advance which unigenes they contained (via Illumina OPA): those are listed in the “expected” column. Column “observed” reports the number of expected unigenes that were actually observed. For the set of expected unigenes, we computed the fraction of those unigenes covered by the contigs (reported in column “AvgExpectedCovg”). The second table/tab (called “U35 unigenes hits by BACS”) shows the same results of BLAST-ing U35 unigenes to BAC assemblies from the viewpoint of unigenes. Here, each row is a U35 unigene: we report the total number of BACs hits by that unigene, the expected number of hits (based on the OPA) and the observed number of hits.(XLSX)

Dataset S5Excel file Dataset_S5 contains the Velvet assembly statistics for each individual pool of 169 BACs for the set HV5 in barley. For each pool, ten choices of 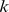-mer were tested in the range [25,79] with a step of 6. The spreadsheet reports only the assembly that maximized the N50. Column titled “K” shows the value of the 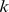-mer in the range which maximized the N50. Column “Cnt” shows the number of contigs produced by Velvet. Column “Used” reports the fraction of the reads used by Velvet. “Med”, “Mean”, “n50”, “max” show the median, mean, n50 and max contig size, respectively. Column “Sum” is the sum of the size of all contigs/scaffolds. “n50/Sz” and “Sum/Sz” report the size of the n50 and the sum over the expected BAC size. “Ns” report the total size of gaps in the scaffolds.(XLSX)

Dataset S6 Excel file Dataset_S6 contains the list of all 3104 solved BAC-unigene relationships. Column A: BAC address in [31] library. First four digits are plate number (0001–0816). Letter is row (A–P). Last two digits are column (01–24). Column B: POPA is SNP namein PilotOPA (1–3) format, as per [27]. Column C: BOPA_C is SNP name in concatenated barley production OPA format, as per [27]. Column D: POPA12_SNP is the original SNP name from the SNP information provider, as per [27]. Column E: POPA3_SNP is the original SNP name from the SNP information provider, as per [27]. Column F: U35 is HarvEST:Barley assembly 35 unigene number corresponding the SNP locus. Column G: MTP is the set of minimal tiling path clones (1–9). Column H: LG_2009 is the linkage group (chromosome) assignment reported on [27]. Column I: cM_2009 is the centiMorgan position reported in [27]. Column J: cM_2011 is the centiMorgan position reported in [27]. Column K: LG_Arm is the linkage group and arm reported in [28]. Column L: Sort indicates the chromosome and arm determined using flow sorted materials as per [28]. Column M: Luo indicates FPC contig of MingCheng Luo on http://phymap.ucdavis.edu/barley/. Column N: Bozdag indicates the compartmentalized FPC contig of Serdar Bozdag on http://phymap.ucdavis.edu/barley/.
(XLSX)

Figure S1 A screenshot of a successful gene detection. In this example, the gene is found in only one BAC. Consequently, seven pools that contains this BAC are positive (blue dots). Morex barley whole genome DNA served as positive control (triplicate, green dots). *E. coli* DNA served as negative control (duplicate, gold dots).
(EPS)

Figure S2 Overlap distribution between rice BACs: the x-axis represents the size of the overlap (in bp), the y-axis the number of BACs with that overlap.
(EPS)

Table S1 Chromosomal distribution of unigenes (assembly #35) contained in BACs (black numbers), and BACs containing unigenes (red numbers), according to GoldenGate assays.
(PDF)

Table S2 Number of rice reads per pool deconvoluted to one, two, or three BACs; the percentage column reports the fraction of the total number of reads that were deconvoluted to at least one BAC, and the total number of correct reads.
(PDF)

Table S3 Number of barley HV3 reads per pool deconvoluted to one, two, or three BACs; the percentage column reports the fraction of the total number of reads that were deconvoluted to at least one BAC.
(PDF)

Table S4 Number of barley HV4 reads per pool deconvoluted to one, two, or three BACs; the percentage column reports the fraction of the total number of reads that were deconvoluted to at least one BAC.
(PDF)

Table S5 Number of barley HV5 reads per pool deconvoluted to one, two, or three BACs; the percentage column reports the fraction of the total number of reads that were deconvoluted to at least one BAC.
(PDF)

Table S6 Number of barley HV6 reads per pool deconvoluted to one, two, or three BACs; the percentage column reports the fraction of the total number of reads that were deconvoluted to at least one BAC.
(PDF)

Video S1 File Video_S1 shows a 75 second video of the manual pooling process. An LCD projector was mounted over a workbench to help with the manual pipetting process. The video is in MPEG-4 format (use Quicktime or VLC to play).
(MP4)

## References

[1] Kircher M, Sawyer S, Meyer M (2011) Double indexing overcomes inaccuracies in multiplex sequencing on the Illumina platform. Nucleic Acids Research 40: e3–e3. 22021376 10.1093/nar/gkr771 PMC3245947

[2] Alon S, Vigneault F, Eminaga S, Christodoulou DC, Seidman JG, et al (2011) Barcoding bias in high-throughput multiplex sequencing of miRNA. Genome Research 21: 1506–1511. 21750102 10.1101/gr.121715.111 PMC3166835

[3] Craig DW, Pearson JV, Szelinger S, Sekar A, Redman M, et al (2008) Identification of genetic variants using bar-coded multiplexed sequencing. Nature Methods 5: 887–93. 18794863 10.1038/nmeth.1251 PMC3171277

[4] Cai WW, Chen R, Gibbs RA, Bradley A (2001) A clone-array pooled strategy for sequencing large genomes. Genome Research 11: 1619–1623. 11591638 10.1101/gr.198101

[5] Csuros M, Milosavljevic A (2002) Pooled genomic indexing (PGI): mathematical analysis and experiment design. In: Proceedings of Workshop on Algorithms in Bioinformatics. LNCS 2452: 10–28.

[6] Csuros M, Li B, Milosavljevic A (2003) Clone-array pooled shotgun mapping and sequencing: design and analysis of experiments. Genome Inform 14: 186–195. 15706533

[7] Milosavljevic A, Harris R, Sodergren E, Jackson A, Kalafus K, et al (2005) Pooled genomic indexing of Rhesus macaque. Genome Research 15: 292–301. 15687293 10.1101/gr.3162505 PMC546531

[8] Prabhu S, Pe'er I (2009) Overlapping pools for high-throughput targeted resequencing. Genome Research 19: 1254–1261. 19447964 10.1101/gr.088559.108 PMC2704440

[9] Erlich Y, Chang K, Gordon A, Ronen R, Navon O, et al (2009) DNA sudoku – harnessing highthroughput sequencing for multiplexed specimen analysis. Genome Research 19: 1243–1253. 19447965 10.1101/gr.092957.109 PMC2704425

[10] Hajirasouliha I, Hormozdiari F, Sahinalp SC, Birol I (2008) Optimal pooling for genome resequencing with ultra-high-throughput short-read technologies. Bioinformatics 24: i32–i40. 18586730 10.1093/bioinformatics/btn173 PMC2718651

[11] Shental N, Amir A, Zuk O (2010) Identification of rare alleles and their carriers using compressed se(que)nsing. Nucleic Acids Research 38: e179–e179. 20699269 10.1093/nar/gkq675 PMC2965256

[12] Erlich Y, Gordon A, Brand M, Hannon GJ, Mitra PP (2010) Compressed genotyping. IEEE Transactions on Information Theory 56: 706–723. 21451737 10.1109/TIT.2009.2037043 PMC3065185

[13] Engler FW, Hatfield J, Nelson W, Soderlund CA (2003) Locating sequence on FPC maps and selecting a minimal tiling path. Genome Research 13: 2152–2163. 12915486 10.1101/gr.1068603 PMC403717

[14] Bozdag S, Close TJ, Lonardi S (2008) Computing the minimal tiling path from a physical map by integer linear programming. In: Proceedings of the Workshop on Algorithms in Bioinformatics. WABI 2008: 148–161.

[15] Thierry-Mieg N (2006) A new pooling strategy for high-throughput screening: the shifted transversal design. BMC Bioinformatics 7: 28. 16423300 10.1186/1471-2105-7-28 PMC1409803

[16] Ding Y, Johnson MD, Chen WQ, Wong D, Chen YJ, et al (2001) Five-color-based highinformation- content fingerprinting of bacterial artificial chromosome clones using type IIS restriction endonucleases. Genomics 74: 142–154. 11386750 10.1006/geno.2001.6547

[17] Luo MC, Thomas C, You FM, Hsiao J, Ouyang S, et al (2003) High-throughput fingerprinting of bacterial artificial chromosomes using the snapshot labeling kit and sizing of restriction fragments by capillary electrophoresis. Genomics 82: 378–389. 12906862 10.1016/s0888-7543(03)00128-9

[18] Soderlund C, Humphray S, Dunham A, French L (2000) Contigs built with fingerprints, markers, and FPC v4.7. Genome Research 10: 1772–1787. 11076862 10.1101/gr.gr-1375r PMC310962

[19] Bozdag S, Close T, Lonardi S (2007) A compartmentalized approach to the assembly of physical maps. In: Proceedings of IEEE International Symposium on Bioinformatics & Bioengineering. BIBE 2007: 218–225.

[20] Du DZ, Hwang FK (1993) Combinatorial Group Testing and Applications. (Series on Applied Mathematics). World Scientific Publishing Company

[21] Du DZ, Hwang F, Wu W, Znati T (2006) New construction for transversal design. Journal of Computational Biology 13: 990–995. 16761923 10.1089/cmb.2006.13.990

[22] Vermeirssen V, Deplancke B, Barrasa MI, Reece-Hoyes JS, Arda HE, et al (2007) Matrix and Steiner-triple-system smart pooling assays for high-performance transcription regulatory network mapping. Nat Meth 4: 659–664. 10.1038/nmeth1063 17589517

[23] Li H, Durbin R (2009) Fast and accurate short read alignment with Burrows-Wheeler transform. Bioinformatics 25: 1754–1760. 19451168 10.1093/bioinformatics/btp324 PMC2705234

[24] Zerbino D, Birney E (2008) Velvet: Algorithms for de novo short read assembly using de Bruijn graphs. Genome Research 8: 821–9. 10.1101/gr.074492.107 PMC2336801 18349386

[25] Li R, Zhu H, Ruan J, Qian W, Fang X, et al (2010) De novo assembly of human genomes with massively parallel short read sequencing. Genome Research 20: 265–272. 20019144 10.1101/gr.097261.109 PMC2813482

[26] Simpson JT, Wong K, Jackman SD, Schein JE, Jones SJ, et al (2009) ABySS: A parallel assembler for short read sequence data. Genome Research 19: 1117–1123. 19251739 10.1101/gr.089532.108 PMC2694472

[27] Close T, Bhat P, Lonardi S, Wu Y, Rostoks N, et al (2009) Development and implementation of high-throughput SNP genotyping in barley. BMC Genomics 10: 582. 19961604 10.1186/1471-2164-10-582 PMC2797026

[28] Muñoz-Amatriaín M, Moscou MJ, Bhat PR, Svensson JT, Bartoš J, et al (2011) An improved consensus linkage map of barley based on flow-sorted chromosomes and single nucleotide polymorphism markers. The Plant Genome 4: 1–12.

[29] Alpert M, Wanamaker S, Duma D, Fenton RD, Ma Y, et al (2011) A genome sequence resource for barley. Barley Genetics Newsletter 41: 10–11.

[30] Wicker T, Zimmermann W, Perovic D, Paterson A, Ganal M, et al (2005) A detailed look at 7 million years of genome evolution in a 439 kb continuous sequence at the barley Hv-eIF4E locus: recombination, rearrangements and repeats. The Plant J 41: 184–194. 15634196 10.1111/j.1365-313X.2004.02285.x

[31] Yu Y, Tomkins JP, Waugh R, Frisch DA, Kudrna D, et al (2000) A bacterial artificial chromosome library for barley (Hordeum vulgare L.) and the identification of clones containing putative resistance genes. Theoretical and Applied Genetics 101: 1093–1099.

[32] Madishetty K, Condamine P, Svensson JT, Rodriguez E, Close TJ (2007) An improved method to identify BAC clones using pooled overgos. Nucleic Acids Research 35: e5. 17151072 10.1093/nar/gkl920 PMC1761434

[33] Fan JB, Chee MS, Gunderson KL (2006) Highly parallel genomic assays. Nat Rev Genet 7: 632–644. 16847463 10.1038/nrg1901

[34] Yang X, Dorman KS, Aluru S (2010) Reptile: representative tiling for short read error correction. Bioinformatics 26: 2526–2533. 20834037 10.1093/bioinformatics/btq468

[35] Treangen TJ, Salzberg SL (2011) Repetitive DNA and next-generation sequencing: computational challenges and solutions. Nature Reviews Genetics 13: 36–46. 10.1038/nrg3117 PMC3324860 22124482

[36] Roach J, Boysen C, Wang K, Hood L (1995) Pairwise end sequencing: a unified approach to genomic mapping and sequencing. Genomics 26: 345–353. 7601461 10.1016/0888-7543(95)80219-c

[37] Weber J, Myers E (1997) Human whole-genome shotgun sequencing. Genome Research 7: 401–409. 9149936 10.1101/gr.7.5.401

[38] Green E (2001) Strategies for the systematic sequencing of complex genomes. Nature Reviews Genetics 2: 573–583. 10.1038/35084503 11483982

[39] Salzberg SL, Phillippy AM, Zimin A, Puiu D, Magoc T, et al (2012) GAGE: A critical evaluation of genome assemblies and assembly algorithms. Genome Research 22: 557–567. 22147368 10.1101/gr.131383.111 PMC3290791

[40] Earl D, Bradnam K, St John J, Darling A, Lin D, et al (2011) Assemblathon 1: A competitive assessment of de novo short read assembly methods. Genome Research 21: 2224–2241. 21926179 10.1101/gr.126599.111 PMC3227110

[41] Haiminen N, Feltus FA, Parida L (2011) Assessing pooled BAC and whole genome shotgun strategies for assembly of complex genomes. BMC Genomics 12: 194. 21496274 10.1186/1471-2164-12-194 PMC3224119

