# Supplementary material for: Combinatorial Pooling Enables Selective Sequencing of the Barley Gene Space
Source: PLoS Comput Biol. 2013 Apr 4;9(4):e1003010. doi: 10.1371/journal.pcbi.1003010 (PMC3617026; doi:10.1371/journal.pcbi.1003010)
Supplement: Table S1 — Chromosomal distribution of unigenes (assembly #35) contained in BACs (black numbers), and BACs containing unigenes (red numbers), according to GoldenGate assays. (PDF) [file pcbi.1003010.s009.pdf]

Chromosome	Short arm	Long arm	Uncertain	Total
1H	52 / 76	140 / 201	38 / 57	230 / 334
2H	114 / 181	211 / 297	2 / 5	327 / 483
3H	80 / 119	195 / 272	0 / 0	275 / 391
4H	74 / 103	132 / 181	1 / 1	207 / 285
5H	68 / 94	263 / 346	2 / 3	333 / 443
6H	77 / 116	124 / 179	0 / 0	201 / 295
7H	146 / 207	126 / 183	0 / 0	272 / 390
Unmapped			1225 / 25	12 / 25
Total	611 / 896	1191 / 1659	55 / 91	1857 / 2646
Unique				1849 / 2541

Table S1: Chromosomal distribution of unigenes (assembly #35) contained in BACs (black numbers), and BACs containing unigenes (red numbers), according to GoldenGate assays.
