# Supplementary material for: Combinatorial Pooling Enables Selective Sequencing of the Barley Gene Space
Source: PLoS Comput Biol. 2013 Apr 4;9(4):e1003010. doi: 10.1371/journal.pcbi.1003010 (PMC3617026; doi:10.1371/journal.pcbi.1003010)
Supplement: Table S2 — Number of rice reads per pool deconvoluted to one, two, or three BACs; the percentage column reports the fraction of the total number of reads that were deconvoluted to at least one BAC, and the total number of correct reads. (PDF) [file pcbi.1003010.s010.pdf]

Rice pool	1 BAC	2 BACs	3 BACs	%Total	%Correct	Rice pool	1 BAC	2 BACs	3 BACs	%Total	%Correct
1	390,925	1,021,202	218,965	81.55%	99.57%	47	415,306	958,780	238,508	80.63%	99.53%
2	401,930	1,010,577	226,119	81.93%	99.45%	48	395,628	988,326	244,361	81.42%	99.39%
3	446,845	1,019,560	199,438	83.29%	99.59%	49	341,453	1,062,420	232,221	81.8%	99.55%
4	460,513	1,012,335	187,729	83.03%	99.59%	50	440,861	943,720	244,218	81.44%	99.55%
5	455,705	947,515	222,519	81.29%	99.52%	51	441,704	983,305	192,133	80.86%	99.55%
6	391,456	1,044,628	198,262	81.72%	99.63%	52	410,569	962,425	242,805	80.79%	99.62%
7	391,010	1,045,500	230,553	83.35%	99.62%	53	400,208	1,018,204	211,243	81.48%	99.53%
8	388,850	991,831	243,614	81.21%	99.58%	54	380,140	969,012	253,479	80.13%	99.49%
9	381,752	975,607	256,767	80.71%	99.58%	55	420,342	1,007,343	211,307	81.95%	99.56%
10	346,968	1,045,075	243,428	81.77%	99.64%	56	449,944	979,593	215,811	82.27%	99.57%
11	394,704	964,910	227,215	79.34%	99.57%	57	393,856	1,060,639	209,274	83.19%	99.55%
12	420,363	936,500	222,287	78.96%	99.49%	58	368,063	1,062,521	224,716	82.77%	99.62%
13	411,143	969,745	239,441	81.02%	99.6%	59	382,411	1,064,979	191,622	81.95%	99.56%
14	386,831	1,028,001	226,478	82.07%	99.62%	60	394,017	992,574	235,947	81.13%	99.62%
15	360,496	1,053,183	245,686	82.97%	99.59%	61	428,393	968,451	234,017	81.54%	99.62%
16	413,108	1,031,143	193,165	81.87%	99.6%	62	511,416	934,536	204,130	82.5%	99.59%
17	426,155	984,613	202,242	80.65%	99.54%	63	323,162	1,019,112	248,197	79.52%	99.41%
18	425,161	972,202	229,742	81.36%	99.55%	64	447,481	936,733	230,762	80.75%	99.64%
19	377,124	993,507	256,679	81.37%	99.62%	65	392,007	968,324	242,245	80.13%	99.58%
20	392,747	1,012,836	216,100	81.08%	99.55%	66	346,148	1,021,575	231,422	79.96%	99.49%
21	358,849	1,016,130	237,873	80.64%	99.49%	67	410,069	922,582	230,421	78.15%	99.38%
22	438,686	998,197	214,066	82.55%	99.55%	68	432,649	952,708	224,847	80.51%	99.56%
23	440,145	959,963	230,235	81.52%	99.61%	69	373,656	983,368	260,897	80.9%	99.61%
24	470,767	970,915	207,429	82.46%	99.48%	70	399,624	1,040,903	203,423	82.2%	99.61%
25	413,950	968,673	229,748	80.62%	99.57%	71	417,006	1,032,484	204,127	82.68%	99.59%
26	380,879	993,435	225,879	80.01%	99.56%	72	430,118	1,002,137	212,012	82.21%	99.51%
27	409,336	1,011,286	204,935	81.28%	99.61%	73	430,159	1,020,881	199,634	82.53%	99.66%
28	413,659	970,618	230,663	80.75%	99.58%	74	389,350	983,376	246,105	80.94%	99.56%
29	478,045	956,413	219,851	82.72%	99.63%	75	485,180	968,794	207,182	83.06%	99.58%
30	437,710	958,018	239,748	81.77%	99.62%	76	427,825	999,602	229,448	82.84%	99.64%
31	312,489	1,051,366	263,548	81.37%	99.59%	77	348,986	1,047,405	238,092	81.72%	99.61%
32	399,797	1,001,191	223,652	81.23%	99.45%	78	394,006	1,008,216	227,993	81.51%	99.57%
33	368,754	1,049,749	209,490	81.4%	99.51%	79	349,668	1,028,294	220,179	79.91%	99.51%
34	394,542	1,029,862	193,241	80.88%	99.54%	80	422,887	975,647	231,153	81.48%	99.46%
35	384,702	1,067,284	185,431	81.87%	99.51%	81	404,990	998,443	222,417	81.29%	99.55%
36	381,991	988,185	228,927	79.96%	99.52%	82	429,214	985,137	219,765	81.71%	99.58%
37	447,843	909,650	263,912	81.07%	99.52%	83	391,829	1,046,567	175,777	80.71%	99.56%
38	453,436	996,709	210,853	83.05%	99.69%	84	418,626	999,325	229,379	82.37%	99.63%
39	403,907	995,495	241,792	82.06%	99.57%	85	412,015	977,566	239,830	81.47%	99.62%
40	383,803	1,010,100	230,899	81.24%	99.56%	86	415,146	973,912	220,785	80.49%	99.43%
41	406,096	998,748	211,387	80.81%	99.53%	87	399,776	980,706	242,806	81.16%	99.57%
42	457,064	1,002,075	188,545	82.38%	99.62%	88	372,785	1,026,350	238,468	81.88%	99.57%
43	430,717	1,028,726	196,809	82.81%	99.6%	89	436,803	988,090	225,036	82.5%	99.63%
44	380,624	1,031,742	210,096	81.12%	99.63%	90	385,299	1,018,026	238,231	82.08%	99.62%
45	357,076	992,491	257,128	80.33%	99.58%	91	445,759	986,738	205,512	81.9%	99.59%
46	426,502	1,023,330	224,662	83.72%	99.62%	**Average**	406,612	998,798	224,167	81.48%	99.57%

Table S2: Number of rice reads per pool deconvoluted to one, two, or three BACs; the percentage column reports the fraction of the total number of reads that were deconvoluted to at least one BAC, and the total number of correct reads.
