# Supplementary material for: Combinatorial Pooling Enables Selective Sequencing of the Barley Gene Space
Source: PLoS Comput Biol. 2013 Apr 4;9(4):e1003010. doi: 10.1371/journal.pcbi.1003010 (PMC3617026; doi:10.1371/journal.pcbi.1003010)
Supplement: Table S3 — Number of barley HV3 reads per pool deconvoluted to one, two, or three BACs; the percentage column reports the fraction of the total number of reads that were deconvoluted to at least one BAC. (PDF) [file pcbi.1003010.s011.pdf]

| HV3 pool | 1 BAC | 2 BACs | 3 BACs | Percentage | HV3 pool | 1 BAC | 2 BACs | 3 BACs | Percentage |
|---|---|---|---|---|---|---|---|---|---|
| 1 | 5,707,665 | 5,397,453 | 203,396 | 69.18% | 47 | 2,654,465 | 2,289,888 | 84,165 | 68.55% |
| 2 | 7,412,182 | 7,224,679 | 208,716 | 71.30% | 48 | 3,451,050 | 3,034,229 | 88,907 | 68.13% |
| 3 | 7,122,035 | 7,032,456 | 210,342 | 71.51% | 49 | 1,679,069 | 1,597,943 | 62,919 | 63.80% |
| 4 | 6,097,505 | 6,210,009 | 308,579 | 70.52% | 50 | 4,024,617 | 3,598,649 | 170,083 | 67.60% |
| 5 | 4,155,937 | 3,753,010 | 153,058 | 70.25% | 51 | 5,253,431 | 6,011,680 | 130,077 | 69.88% |
| 6 | 7,111,109 | 6,378,901 | 193,988 | 70.95% | 52 | 3,919,110 | 3,641,468 | 86,037 | 69.01% |
| 7 | 3,716,854 | 3,162,972 | 105,988 | 71.06% | 53 | 4,133,773 | 4,407,511 | 168,756 | 69.96% |
| 8 | 5,241,445 | 4,603,032 | 218,994 | 71.20% | 54 | 6,220,472 | 6,084,451 | 280,569 | 68.81% |
| 9 | 5,039,067 | 5,595,192 | 198,750 | 68.11% | 55 | 8,028,682 | 7,523,656 | 220,713 | 69.20% |
| 10 | 2,959,104 | 2,617,033 | 97,239 | 62.37% | 56 | 3,172,903 | 2,982,963 | 80,297 | 68.34% |
| 11 | 3,770,578 | 3,857,924 | 118,078 | 71.48% | 57 | 4,080,858 | 3,944,534 | 130,627 | 68.24% |
| 12 | 3,515,447 | 3,738,271 | 82,089 | 69.11% | 58 | 2,654,715 | 2,373,371 | 51,726 | 70.42% |
| 13 | 6,888,454 | 6,009,571 | 187,779 | 65.26% | 59 | 7,410,276 | 6,620,099 | 195,760 | 69.98% |
| 14 | 2,869,142 | 3,224,217 | 124,179 | 70.32% | 60 | 5,273,265 | 4,776,469 | 153,016 | 69.10% |
| 15 | 2,966,197 | 2,881,219 | 135,284 | 65.74% | 61 | 2,206,444 | 2,264,909 | 39,501 | 70.78% |
| 16 | 2,867,964 | 2,590,355 | 101,086 | 70.91% | 62 | 3,546,518 | 3,535,025 | 83,438 | 70.30% |
| 17 | 2,964,886 | 2,791,077 | 115,930 | 65.80% | 63 | 4,995,703 | 5,125,251 | 273,626 | 71.58% |
| 18 | 2,648,327 | 2,476,321 | 73,874 | 65.04% | 64 | 4,454,925 | 3,934,635 | 214,741 | 69.55% |
| 19 | 4,267,383 | 3,810,933 | 107,386 | 65.56% | 65 | 7,211,674 | 6,598,179 | 284,570 | 69.36% |
| 20 | 3,569,784 | 2,928,244 | 73,463 | 70.21% | 66 | 5,477,224 | 5,734,381 | 145,932 | 68.30% |
| 21 | 3,045,115 | 2,865,862 | 58,031 | 69.95% | 67 | 5,368,020 | 4,420,260 | 103,563 | 69.25% |
| 22 | 4,225,245 | 4,197,622 | 122,568 | 71.31% | 68 | 3,496,852 | 3,066,728 | 107,433 | 69.33% |
| 23 | 4,148,662 | 3,954,947 | 194,786 | 72.48% | 69 | 2,974,337 | 2,678,500 | 112,662 | 71.39% |
| 24 | 2,859,240 | 2,635,102 | 75,003 | 66.67% | 70 | 6,680,195 | 6,514,717 | 258,672 | 71.14% |
| 25 | 2,677,318 | 2,503,604 | 65,904 | 61.71% | 71 | 1,899,443 | 2,076,722 | 70,476 | 68.99% |
| 26 | 4,152,115 | 4,188,072 | 199,465 | 65.83% | 72 | 2,131,326 | 2,091,950 | 106,565 | 70.09% |
| 27 | 4,870,382 | 5,131,352 | 122,033 | 70.14% | 73 | 2,209,899 | 2,327,000 | 86,573 | 70.14% |
| 28 | 4,601,120 | 4,690,423 | 129,649 | 64.06% | 74 | 2,339,443 | 2,476,306 | 110,601 | 68.16% |
| 29 | 1,496,992 | 1,575,520 | 55,813 | 63.11% | 75 | 4,486,611 | 3,584,594 | 75,017 | 71.05% |
| 30 | 689,050 | 638,725 | 25,079 | 42.66% | 76 | 3,339,194 | 3,199,938 | 113,511 | 69.30% |
| 31 | 2,773,501 | 2,462,944 | 74,316 | 69.45% | 77 | 2,345,758 | 2,070,358 | 81,622 | 69.27% |
| 32 | 2,177,643 | 1,821,027 | 81,966 | 63.97% | 78 | 3,131,958 | 3,295,655 | 69,911 | 68.57% |
| 33 | 3,069,249 | 3,074,086 | 135,475 | 66.66% | 79 | 4,250,828 | 3,754,936 | 142,775 | 70.85% |
| 34 | 1,862,398 | 1,842,243 | 59,437 | 65.93% | 80 | 2,788,524 | 2,870,574 | 73,870 | 69.56% |
| 35 | 2,952,894 | 2,592,244 | 67,450 | 68.33% | 81 | 3,971,309 | 3,470,064 | 67,215 | 68.97% |
| 36 | 3,005,815 | 2,579,780 | 77,383 | 70.57% | 82 | 5,794,972 | 5,722,724 | 183,505 | 65.00% |
| 37 | 3,752,411 | 3,806,305 | 106,054 | 68.19% | 83 | 3,867,556 | 3,557,021 | 115,237 | 69.05% |
| 38 | 3,540,393 | 3,709,472 | 171,186 | 69.19% | 84 | 3,635,943 | 3,649,551 | 145,271 | 67.27% |
| 39 | 2,720,022 | 2,511,969 | 91,898 | 65.81% | 85 | 6,249,613 | 5,734,101 | 226,693 | 69.20% |
| 40 | 1,648,459 | 1,704,868 | 48,074 | 64.46% | 86 | 4,729,766 | 4,120,351 | 179,666 | 71.00% |
| 41 | 2,137,004 | 2,082,788 | 96,377 | 58.68% | 87 | 7,677,653 | 6,591,320 | 210,415 | 69.56% |
| 42 | 2,818,344 | 2,440,948 | 69,630 | 68.49% | 88 | 3,095,337 | 3,005,906 | 103,344 | 70.00% |
| 43 | 3,875,895 | 4,089,128 | 125,436 | 65.50% | 89 | 4,785,482 | 4,895,828 | 112,572 | 69.46% |
| 44 | 4,114,272 | 4,373,857 | 170,411 | 63.66% | 90 | 3,137,981 | 3,525,446 | 141,496 | 66.61% |
| 45 | 5,493,829 | 4,722,889 | 256,573 | 65.70% | 91 | 5,279,639 | 5,057,788 | 240,976 | 69.19% |
| 46 | 6,639,573 | 6,396,366 | 173,031 | 68.85% | **Average** | 4,019,284 | 3,832,007 | 132,487 | 68.14% |

**Table S3:** Number of barley HV3 reads per pool deconvoluted to one, two, or three BACs; the percentage column reports the fraction of the total number of reads that were deconvoluted to at least one BAC.
