# Supplementary material for: Combinatorial Pooling Enables Selective Sequencing of the Barley Gene Space
Source: PLoS Comput Biol. 2013 Apr 4;9(4):e1003010. doi: 10.1371/journal.pcbi.1003010 (PMC3617026; doi:10.1371/journal.pcbi.1003010)
Supplement: Table S4 — Number of barley HV4 reads per pool deconvoluted to one, two, or three BACs; the percentage column reports the fraction of the total number of reads that were deconvoluted to at least one BAC. (PDF) [file pcbi.1003010.s012.pdf]

HV4 pool	1 BAC	2 BACs	3 BACs	Percentage	HV4 pool	1 BAC	2 BACs	3 BACs	Percentage
1	2,444,998	186,846	4,846	56.53%	47	3,985,884	247,631	4,817	58.01%
2	3,191,634	179,316	4,920	58.41%	48	3,221,468	205,179	5,607	54.32%
3	3,400,458	219,185	3,640	58.88%	49	2,787,022	168,102	4,868	56.45%
4	3,163,560	246,824	4,115	59.55%	50	2,823,380	202,424	3,353	59.07%
5	3,791,889	263,748	4,919	59.51%	51	2,835,675	201,401	2,967	61.65%
6	2,494,259	156,940	2,827	60.35%	52	3,202,268	219,463	3,800	61.47%
7	4,213,107	241,538	3,481	62.09%	53	929,864	77,267	2,291	50.62%
8	2,727,738	194,901	3,241	61.49%	54	2,264,874	124,650	2,077	63.26%
9	3,139,692	219,764	3,406	59.09%	55	5,112,864	375,480	6,125	63.01%
10	1,664,334	121,685	2,547	54.74%	56	3,457,029	229,632	2,872	64.08%
11	2,397,626	204,570	3,407	57.71%	57	3,220,227	254,637	3,498	61.04%
12	3,122,190	182,096	3,310	56.83%	58	5,167,202	301,109	6,687	60.94%
13	1,433,395	101,727	2,913	53.42%	59	9,882,661	696,919	13,262	62.42%
14	2,577,358	175,926	3,212	59.17%	60	3,385,523	219,574	4,691	59.33%
15	5,062,673	321,267	5,839	60.60%	61	6,307,320	393,875	8,795	59.44%
16	2,449,057	156,550	2,413	59.50%	62	2,024,036	138,078	2,486	55.70%
17	2,848,307	227,831	3,808	58.56%	63	3,125,175	210,489	3,082	61.84%
18	3,267,632	234,540	3,478	61.47%	64	3,563,068	273,996	4,647	60.77%
19	2,774,069	180,131	3,875	59.94%	65	2,293,455	150,765	2,565	61.33%
20	2,976,448	250,513	3,862	59.19%	66	5,192,715	363,305	5,550	60.07%
21	2,745,834	175,739	2,840	60.75%	67	4,020,101	271,898	4,645	59.44%
22	2,117,718	134,527	2,094	58.33%	68	1,970,211	140,740	2,171	61.98%
23	3,946,114	273,298	5,689	58.53%	69	1,907,448	148,700	2,391	60.24%
24	2,156,654	123,769	3,549	57.06%	70	5,410,990	306,584	5,988	60.39%
25	4,894,252	358,875	10,690	57.64%	71	6,057,189	391,079	7,722	58.16%
26	4,583,395	321,028	5,748	59.66%	72	2,414,375	159,354	3,573	57.30%
27	5,416,702	385,838	4,973	63.63%	73	6,294,220	382,276	8,193	59.23%
28	3,371,029	242,269	4,336	59.74%	74	5,011,232	385,339	9,471	60.44%
29	2,830,436	203,936	3,812	60.20%	75	4,757,550	384,145	7,872	60.23%
30	2,963,694	194,605	3,332	62.83%	76	3,748,138	260,044	4,434	61.07%
31	2,997,871	176,607	2,692	62.33%	77	6,722,989	509,013	9,600	60.63%
32	3,159,149	239,780	4,069	60.32%	78	3,692,585	227,229	2,785	62.21%
33	2,501,427	173,286	2,958	59.72%	79	4,221,229	323,784	5,924	59.70%
34	4,144,441	253,017	4,625	59.87%	80	4,796,680	358,319	7,376	60.51%
35	6,557,247	446,096	9,428	59.50%	81	4,126,889	281,664	4,604	62.72%
36	5,123,594	341,055	7,412	57.96%	82	4,427,851	276,944	4,953	59.43%
37	3,069,860	192,975	7,212	50.42%	83	4,295,483	285,709	5,708	59.06%
38	4,420,677	275,457	5,055	60.77%	84	5,199,837	361,929	7,028	59.03%
39	4,401,318	319,103	5,251	60.88%	85	6,199,370	419,074	7,818	57.28%
40	2,901,643	181,728	2,340	60.35%	86	4,269,213	277,515	3,990	59.00%
41	4,430,747	361,196	6,102	61.37%	87	4,587,985	337,798	6,988	59.15%
42	3,650,796	236,604	3,480	63.76%	88	5,188,447	348,467	7,113	61.71%
43	2,908,849	216,611	4,934	61.87%	89	3,879,399	272,732	5,155	62.46%
44	3,301,821	247,484	4,119	61.60%	90	4,659,411	334,941	5,294	61.89%
45	4,728,567	334,227	4,968	60.58%	91	3,987,717	323,273	5,669	60.44%
46	2,853,964	158,524	3,169	59.71%	**Average**	3,757,653	257,758	4,829	59.91%

Table S4: Number of barley HV4 reads per pool deconvoluted to one, two, or three BACs; the percentage column reports the fraction of the total number of reads that were deconvoluted to at least one BAC.
