# Supplementary material for: Combinatorial Pooling Enables Selective Sequencing of the Barley Gene Space
Source: PLoS Comput Biol. 2013 Apr 4;9(4):e1003010. doi: 10.1371/journal.pcbi.1003010 (PMC3617026; doi:10.1371/journal.pcbi.1003010)
Supplement: Table S5 — Number of barley HV5 reads per pool deconvoluted to one, two, or three BACs; the percentage column reports the fraction of the total number of reads that were deconvoluted to at least one BAC. (PDF) [file pcbi.1003010.s013.pdf]

HV5 pool	1 BAC	2 BACs	3 BACs	Percentage	HV5 pool	1 BAC	2 BACs	3 BACs	Percentage
1	4,819,809	1,361,096	22,413	72.23%	47	2,678,468	707,740	11,405	70.71%
2	3,356,009	773,496	11,241	70.92%	48	1,369,486	346,430	4,368	69.21%
3	2,928,866	852,784	11,806	73.49%	49	3,487,998	1,020,879	11,639	69.47%
4	2,719,426	655,721	9,354	70.87%	50	4,152,521	1,011,419	16,806	70.53%
5	2,134,539	550,159	6,554	72.34%	51	2,570,124	712,912	8,537	71.10%
6	2,621,358	740,313	9,647	71.23%	52	2,343,768	674,626	9,985	72.12%
7	2,296,717	684,713	9,099	70.07%	53	3,346,147	746,610	11,218	71.17%
8	2,159,752	621,167	7,954	71.77%	54	4,435,090	1,157,021	15,397	70.77%
9	2,433,324	674,402	9,906	70.94%	55	3,447,887	908,114	12,100	72.87%
10	2,495,663	665,023	8,358	69.78%	56	2,469,629	702,317	9,724	71.93%
11	4,487,909	1,139,795	14,919	71.93%	57	4,482,223	1,265,902	19,130	73.73%
12	2,806,989	656,867	7,383	70.85%	58	3,830,086	1,014,000	14,148	73.47%
13	5,583,911	1,347,428	23,039	70.88%	59	2,614,274	698,170	10,303	72.59%
14	2,284,650	595,029	9,435	69.37%	60	3,768,648	1,087,327	13,907	72.18%
15	2,857,396	635,683	13,903	70.90%	61	3,533,073	955,348	12,618	73.50%
16	2,057,522	520,255	6,564	73.22%	62	2,066,678	561,530	6,632	72.20%
17	2,384,207	578,633	7,367	69.53%	63	2,843,156	753,623	9,946	73.03%
18	3,898,797	1,037,323	14,261	71.89%	64	2,824,251	749,455	11,829	72.14%
19	4,070,134	1,029,797	15,535	71.86%	65	4,306,217	1,002,165	15,848	70.85%
20	2,596,261	631,889	9,508	72.99%	66	2,543,969	636,442	9,883	72.46%
21	3,413,895	928,178	12,544	71.79%	67	3,278,529	802,352	11,650	72.48%
22	2,730,569	760,841	9,474	71.92%	68	5,067,615	1,421,635	17,796	72.31%
23	4,015,312	1,051,903	12,655	70.61%	69	3,133,900	752,926	9,632	74.18%
24	3,134,394	917,029	12,535	70.46%	70	5,066,050	1,364,484	23,341	72.79%
25	2,544,742	691,088	7,881	70.20%	71	4,984,935	1,405,677	20,345	71.14%
26	2,520,794	676,274	8,294	71.63%	72	3,671,420	974,582	12,897	71.74%
27	1,450,596	261,775	3,390	69.43%	73	3,935,179	1,049,687	13,507	71.70%
28	3,102,789	477,918	10,209	70.81%	74	2,667,700	735,036	13,378	72.22%
29	1,560,722	429,857	5,518	72.62%	75	1,891,923	446,900	5,392	72.60%
30	2,251,092	632,236	8,219	72.85%	76	6,189,933	1,808,781	21,952	70.68%
31	2,057,050	616,615	8,083	72.61%	77	5,311,495	1,133,613	13,325	71.11%
32	2,492,383	753,568	11,370	72.59%	78	3,512,563	919,104	13,369	70.25%
33	1,853,107	563,378	6,254	72.01%	79	2,730,042	524,566	7,215	69.40%
34	4,015,683	1,051,878	18,413	72.18%	80	2,778,343	506,951	7,824	70.41%
35	1,902,102	642,667	8,702	70.89%	81	3,693,776	1,088,443	12,793	72.51%
36	1,316,800	359,575	4,450	67.32%	82	4,184,258	1,219,432	17,039	73.09%
37	3,475,027	1,089,877	13,217	72.35%	83	2,354,926	640,439	12,482	73.17%
38	4,375,684	1,084,768	15,712	69.94%	84	5,551,482	1,148,488	14,139	70.54%
39	3,702,940	893,985	11,375	72.63%	85	2,858,149	844,537	10,748	72.93%
40	3,061,833	916,000	14,896	71.10%	86	3,202,475	912,971	13,387	73.88%
41	3,240,123	761,146	8,966	71.34%	87	2,346,634	665,816	10,151	72.80%
42	2,284,961	625,217	9,521	72.03%	88	2,861,075	710,085	7,662	73.25%
43	2,247,624	546,920	6,379	71.41%	89	4,954,952	1,273,423	18,529	72.17%
44	1,789,146	460,577	5,713	73.06%	90	2,591,877	764,120	8,811	72.61%
45	1,809,105	438,958	5,845	71.91%	91	2,623,489	834,179	11,398	71.98%
46	1,481,743	401,135	10,960	37.63%	**Average**	3,114,064	818,079	11,462	71.30%

Table S5: Number of barley HV5 reads per pool deconvoluted to one, two, or three BACs; the percentage column reports the fraction of the total number of reads that were deconvoluted to at least one BAC.
