# Supplementary material for: Combinatorial Pooling Enables Selective Sequencing of the Barley Gene Space
Source: PLoS Comput Biol. 2013 Apr 4;9(4):e1003010. doi: 10.1371/journal.pcbi.1003010 (PMC3617026; doi:10.1371/journal.pcbi.1003010)
Supplement: Table S6 — Number of barley HV6 reads per pool deconvoluted to one, two, or three BACs; the percentage column reports the fraction of the total number of reads that were deconvoluted to at least one BAC. (PDF) [file pcbi.1003010.s014.pdf]

HV6 pool	1 BAC	2 BACs	3 BACs	Percentage	HV6 pool	1 BAC	2 BACs	3 BACs	Percentage
1	1,675,358	419,696	8,906	66.02%	47	2,227,556	613,632	7,898	65.41%
2	3,280,877	841,336	15,239	68.73%	48	1,011,015	240,566	2,961	49.70%
3	2,498,889	592,965	10,641	67.12%	49	865,169	220,554	2,842	44.10%
4	1,449,635	350,015	4,755	63.65%	50	467,035	132,642	2,361	38.59%
5	1,868,230	426,423	5,739	65.49%	51	438,858	123,850	2,752	45.25%
6	1,658,743	447,310	7,960	65.35%	52	933,725	266,009	3,432	55.00%
7	2,340,090	636,303	8,845	65.70%	53	723,768	166,032	3,829	50.79%
8	2,469,401	684,043	10,499	67.84%	54	679,861	157,209	4,230	50.38%
9	2,632,014	752,788	11,759	61.73%	55	2,019,753	533,543	7,763	64.86%
10	2,318,027	588,209	8,871	62.61%	56	1,004,255	236,920	3,680	52.39%
11	2,559,914	721,148	9,798	63.36%	57	1,259,837	307,417	4,341	57.19%
12	1,931,664	535,362	9,026	66.14%	58	1,628,323	474,164	7,343	56.95%
13	761,467	179,943	3,415	62.00%	59	1,040,004	272,182	4,357	51.08%
14	1,409,398	358,983	8,444	63.77%	60	1,054,348	279,007	5,118	34.79%
15	1,412,044	354,333	7,713	62.12%	61	1,345,434	365,752	5,034	41.41%
16	3,313,577	921,590	11,435	67.80%	62	1,507,283	392,488	4,906	54.31%
17	1,743,091	382,501	6,338	59.23%	63	1,880,849	541,424	6,778	45.40%
18	2,215,377	563,700	7,513	68.36%	64	1,827,831	496,809	6,822	52.77%
19	2,506,595	625,598	11,515	63.60%	65	1,024,303	210,909	4,070	50.07%
20	1,179,339	282,175	5,041	58.17%	66	2,156,379	542,283	11,676	63.55%
21	1,318,471	318,871	6,875	58.90%	67	1,670,692	405,837	10,701	60.20%
22	1,546,387	381,570	6,391	62.77%	68	1,388,193	402,025	5,451	62.02%
23	1,387,917	363,294	5,033	61.54%	69	1,785,526	393,847	6,785	56.93%
24	2,490,387	647,886	10,161	60.40%	70	1,014,501	279,843	3,859	54.75%
25	1,273,930	364,774	4,584	59.81%	71	2,142,934	619,672	11,284	58.60%
26	3,090,614	803,808	14,544	65.66%	72	1,395,454	346,551	4,954	51.32%
27	1,591,790	393,233	8,756	63.47%	73	924,842	218,220	3,171	55.03%
28	2,650,578	415,119	14,415	60.24%	74	1,392,318	359,081	7,536	60.54%
29	2,626,659	508,097	11,326	63.75%	75	883,823	215,201	2,636	44.10%
30	1,332,029	318,377	6,887	47.95%	76	1,289,184	317,579	3,772	56.67%
31	1,816,375	498,064	8,022	60.23%	77	2,009,751	492,243	9,752	61.35%
32	1,624,935	435,086	5,357	59.62%	78	993,551	283,554	4,414	54.37%
33	1,542,975	461,706	7,538	57.94%	79	1,482,216	351,469	6,570	58.72%
34	1,681,857	476,627	8,673	61.93%	80	1,739,787	387,055	6,905	59.72%
35	1,007,176	292,825	3,587	60.77%	81	990,743	257,318	3,898	59.09%
36	1,369,906	370,653	4,744	58.12%	82	1,488,563	419,669	7,778	44.57%
37	700,677	196,129	2,910	44.62%	83	1,634,853	403,205	5,795	57.54%
38	2,469,565	646,915	9,671	61.25%	84	1,215,412	332,149	6,058	55.59%
39	3,114,938	718,647	9,427	65.20%	85	919,486	242,445	3,521	45.19%
40	2,099,824	493,992	7,798	65.96%	86	1,701,437	414,251	5,371	59.25%
41	1,311,238	248,933	5,583	53.35%	87	888,499	241,684	4,527	52.19%
42	1,623,143	438,055	6,970	60.88%	88	885,919	229,125	5,069	39.96%
43	799,939	182,437	2,529	50.15%	89	1,676,685	392,835	7,138	50.31%
44	1,245,376	325,556	5,870	53.17%	90	751,282	178,759	2,611	36.76%
45	941,502	250,322	5,168	48.23%	91	1,009,777	271,326	4,221	55.32%
46	677,677	165,372	2,785	42.75%	**Average**	1,570,666	400,100	6,649	58.06%

Table S6: Number of barley HV6 reads per pool deconvoluted to one, two, or three BACs; the percentage column reports the fraction of the total number of reads that were deconvoluted to at least one BAC.
